# Supplementary material for: Macromolecular crowding and supersaturation protect hemodialysis patients from the onset of dialysis-related amyloidosis
Source: Nat Commun. 2022 Oct 3;13:5689. doi: 10.1038/s41467-022-33247-3 (PMC9530240; doi:10.1038/s41467-022-33247-3)
Supplement: Supplementary file 1 — Supplementary Information [file 41467_2022_33247_MOESM1_ESM.pdf]

Supplementary Information for

Macromolecular crowding and supersaturation protect hemodialysis patients from the onset of dialysis-related amyloidosis

Authors

Kichitaro Nakajima[a,1,2], Keiichi Yamaguchi[a,1,2], Masahiro Noji[b,1], César Aguirre[c], Kensuke Ikenaka[c], Hideki Mochizuki[c], Lianjie Zhou[d], Hirotsugu Ogi[d], Toru Ito[e], Ichiei Narita[e], Fumitake Gejyo[f], Hironobu Naiki[g], Suguru Yamamoto[e,3a], and Yuji Goto[a,2,3b]

Affiliations

[a]Global Center for Medical Engineering and Informatics, Osaka University, Suita, Osaka 565-0871, Japan;
[b]Graduate School of Human and Environmental Studies, Kyoto University, Yoshidahonmatsu-cho, Sakyo-ku, Kyoto 606-8316, Japan;
[c]Department of Neurology, Graduate School of Medicine, Osaka University, Suita, Osaka 565-0871, Japan;
[d]Graduate School of Engineering, Osaka University, Suita, Osaka 565-0871, Japan;
[e]Division of Clinical Nephrology and Rheumatology, Graduate School of Medical and Dental Sciences, Niigata University, Niigata 951-8510, Japan;
[f]Niigata University of Pharmacy and Applied Life Sciences, Niigata 956-8603, Japan;
[g]Faculty of Medical Sciences, University of Fukui, Fukui 910-1193, Japan.

1: K. N., K. Y., and M. N. contributed equally.

2: Present affiliation: Graduate School of Engineering, Osaka University, Suita, Osaka 565-0871, Japan.

3: Corresponding authors

a: Suguru Yamamoto, yamamotos@med.niigata-u.ac.jp

b: Yuji Goto, gtyj8126@protein.osaka-u.ac.jp

Items contained in this Supplementary Information

Supplementary Notes 1-11

Supplementary Figures 1-14

Supplementary Tables 1-5

Supplementary References

Supplementary Text

Supplementary Notes

Supplementary Note 1: High performance reversed-phase chromatography of aggregates formed by ultrasonication

High performance reversed-phase chromatographic analysis was performed to confirm that the aggregates formed by ultrasonication consisted of intact monomers that had not been fragmented by ultrasonication. The dissolution of preformed aggregates with 90% (v/v) DMSO under acidic conditions was confirmed using thioflavin-T (ThT) dye by spectrofluorometer (F-7100, HITACHI) using FL solutions software (Version 4.2, HITACHI), as shown in Supplementary Fig. 3a. The intact monomer without ultrasonication was prepared as a control. Supplementary Fig. 3b shows the chromatograms of each sample. The amyloid fibrils dissolved by 90% DMSO (acidic) showed the same elution peak as the intact monomer at 15.76 min. Enlarged chromatograms are shown in the main body in Fig. 1d. The chromatogram of the dissolved amyloid fibrils showed a sub-peak, as presented in Supplementary Fig. 3c, but the area under the sub-peak curve was 1.8%, which was negligible compared with the area under the main peak. These results demonstrated that amyloid fibrils formed by ultrasonication consisted of intact monomers without any ultrasonic fragmentations or other damage during the ultrasonication-based assay.

Supplementary Note 2: Supplementary TEM images

To confirm the reproducibility of the TEM images of the main body (Fig. 1e-g), multiple images acquired with the same sample were included as supplementary data, as shown in Supplementary Fig. 4. As in the TEM images of the main body, the ThT-

positive aggregates formed by ultrasonication showed a weak-contrast fibrillar morphology (Supplementary Fig. 4a), and the aggregates seeded and elongated from the amyloid fibrils formed by ultrasonication showed a clear-contrast fibrous morphology (Supplementary Fig. 4b). The ThT-negative aggregates formed in the presence of 15% (v/v) serum were amorphous (Supplementary Fig. 4c).

Supplementary Note 3: Serum concentration of β2m monomer in each patient cohort

Serum samples were collected from non-dialysis controls (DT(-), N = 30) and dialysis patients (DT(+), N = 58). In the group of dialysis patients, 28 patients donated two serum samples, one collected immediately before (DT(+, Pre)) and one immediately after (DT(+, Post)) a maintenance dialysis treatment. The remaining 30 patients donated their serum samples collected immediately before (DT(+, Pre)) a maintenance dialysis treatment. Thus, 116 serum samples were used in this study.

For all sera, the concentrations of β2m monomer were measured with enzyme linked immunosorbent assay (ELISA) using a commercially available reagent kit (KGE019, Human beta 2-Microglobulin Parameter Assay Kit, R&D Systems). The results of quantification for all sera are shown in Supplementary Fig. 5.

The serum concentrations of β2m monomer were clearly higher in the serum of dialysis patients than those of non-dialysis controls (Supplementary Fig. 5a). The concentrations in dialysis patients and non-dialysis controls were 23.9 ± 5.2 (N = 30) and 1.3 ± 0.4 μg/mL (N = 30), respectively, which were consistent with the literature [1,2]. Meanwhile, the serum concentrations of β2m monomer markedly decreased after maintenance dialysis treatments (Supplementary Fig. 5b). The pre- and post-dialysis β2m concentrations were 24.9 ± 5.3 (N = 28) and 7.7 ± 4.4 μg/mL (N = 28), respectively,

which were also consistent with the literature [1,2]. When serum was added to the recombinant β2m solution at a concentration of 5% (v/v), the concentration of β2m monomer from sera was at most 2 μg/mL, being much less than in standard solutions (1.0 mg/mL) for the HANABI assay, and its effects on amyloid fibril formation would be negligible.

Supplementary Note 4: Relationship between ThT fluorescence intensity and the amount of amyloid fibrils in the presence of sera

In amyloid fibril formation, the addition of preformed fibrils bypasses the primary nucleation and accelerates amyloid formation, the so-called seeding reaction [3]. In general, the reaction time for amyloid formation is shortened in a seed-concentration-dependent manner, i.e., the higher seed concentrations induce more rapid amyloid fibril formation [4]. Based on the concentration-dependent manner of the seeding reaction, we qualitatively evaluated the relationship between the ThT fluorescence intensity of amyloid fibrils formed at various serum concentrations and the net amount of amyloid fibrils.

As shown in Supplementary Fig. 6a, b, the lag time of amyloid formation depended on the seed concentration, indicating that the shorter the lag time, the greater the amount of preformed amyloid fibrils in the seed solution. Next, the seeds formed at serum concentrations of 0-10% (v/v) were added to the monomer solution, and the concentration of amyloid fibrils was evaluated by the seeding reaction, as shown in Supplementary Fig. 6c. As summarized in Supplementary Fig. 6d, the higher serum concentration led to a longer lag time in the seeding reaction. Supplementary Fig. 6e shows the relationship between ThT fluorescence intensity and the lag time in the seeding reaction. A shorter lag time indicates a higher amyloid fibril concentration in the

sample solution, which means that the fibril concentration in the sample solution positively correlates with ThT fluorescence intensity of the sample solution.

Supplementary Note 5: Examination of significance of change in the effects of serum on amyloid formation before and after maintenance dialysis treatments

To examine the change in the effects of serum on amyloid fibril formation before and after maintenance dialysis treatment, the ThT fluorescence assay with ultrasonication was performed using sera collected from 28 patients immediately before and after maintenance dialysis treatments. ThT kinetics are shown in Supplementary Fig. 8a, where red and blue lines denote the results with sera collected before and after the treatment, respectively. For each sample, ThT kinetics were measured using multiple independent solutions ($n \geq 4$). Using the data, the significance of any change in the lag time and ThT fluorescence intensity between sera collected before and after maintenance dialysis treatments was investigated by the unpaired one-sided *t*-test, as shown in Supplementary Fig. 8b, c, respectively. In 28 patients examined, 16 and 23 patients showed significantly slower kinetics (lag time) and less resultant amyloid fibrils (ThT fluorescence intensity) after the maintenance dialysis treatments, indicating that the inhibitory effects of serum on fibril formation improved after a single dialysis treatment in more than half of the dialysis patients (Supplementary Table 1 and 2). In the main text, the results of the paired one-sided *t*-test for each average value are shown in Fig. 4c, d.

Supplementary Note 6: Correlation between serum components and β2m fibril formation

The correlation of each serum component with the amyloid formation was analyzed by means of the Pearson correlation coefficient. The concentrations of 27

serum components were measured for each serum, their concentrations were plotted on the horizontal axis, and the lag time and ThT fluorescence intensity were plotted on the vertical axes, shown in Supplementary Fig. 9. For each plot, the absolute value of the Pearson correlation coefficient, |*r*|, was calculated, and is shown in the inset of Supplementary Fig. 9. The top 10 components with the highest correlation coefficients are summarized in Supplementary Tables 3 and 4. Among them, we focused on five serum components that showed correlation coefficients of 0.5 or higher for both the lag time and ThT fluorescence intensity: β2m monomer, blood urea nitrogen, creatinine, ureic acid, and serum albumin. Here, it should be noted that the final concentration of β2m monomers was negligible because it was less than 1% of the concentration of recombinant β2m monomers included in the standard solution used in the HANABI assays. On the other hand, blood urea nitrogen is an indicator of the blood concentration of urea. Urea is a classical denaturant of native proteins [5], shifting the equilibrium from native folded monomers to denatured unfolded monomers in a concentration-dependent manner. We previously studied the urea-induced unfolding of β2m monomers under a neutral condition [6]. The results showed that the urea concentration at the midpoint of denaturation was ~5 M. The serum concentration of blood urea nitrogen was less than 100 mg/dL (Supplementary Fig. 9, panel 25), which corresponds to a urea concentration of less than 36 mM. Such a low-concentration urea fails to affect the β2m monomer stability and its amyloid fibril formation. Therefore, since the high correlation between these two serum components and amyloidogenecity can be attributed to a spurious correlation, these two serum components were excluded as candidates for factors affecting amyloid formation.

Effects of three other serum components: serum albumin, ureic acid, and creatinine, on β2m amyloid fibril formation were investigated, as shown in Supplementary

Fig. 10. For the experiments, serum albumin, ureic acid, and creatinine were added to the β2m monomer solution with a monomer concentration of 0.3 mg/mL including 20 mM sodium phosphate (pH 7.4), 500 mM NaCl, and 5 μM ThT dye. The results showed that serum albumin markedly supressed β2m fibril formation in a concentration-dependent manner (Supplementary Fig. 10d), while ureic acid (Supplementary Fig. 10h) and creatinine (Supplementary Fig. 10l) did not.

Supplementary Note 7: Near-UV CD spectroscopy

To observe the denaturation curve of β2m monomer in the presence of serum albumin, the near-UV CD spectra were measured. The native β2m monomer possesses a unique peak at 293.5 nm (Supplementary Fig. 12a), but not for serum albumin at 280-300 nm (Supplementary Fig. 12b). In the thermal denaturation experiment, β2m monomer showed a cooperative denaturation with the denaturation midpoint of 66.5 °C (Supplementary Fig. 12c) at a wavelength of 293.5 nm, showing reasonable agreement with one acquired by the far-UV CD spectrum (Fig. 1c). Although the cooperative denaturation of serum albumin was observed by far-UV CD spectroscopy at a wavelength of 225 nm (Supplementary Fig. 12d), it was not observed (Supplementary Fig. 12c) at a wavelength of 293.5 nm. These results indicate that measuring CD ellipticity at 293.5 nm allows us to extract the denaturation curve of β2m monomer in the presence of serum albumin. We adopted this wavelength to evaluate the thermostability of β2m monomer in the presence of serum albumin at various concentrations (Fig. 5h).

Supplementary Note 8: Determination of the solubility of denatured β2m monomers under neutral conditions

The β2m monomer concentration in the supernatant of the solution after amyloid fibril formation was measured as described in Methods. We confirmed that β2m monomers did not precipitate under the ultracentrifugation conditions (100,000x*g* for 1 h) at 37 °C. The monomer concentration in the supernatant of sample solutions after the HANABI assay was determined to be 772 ± 389 μg/L (~65.4 nM), which is the concentration of the native monomers ($[N]^E$) because the anti-β2m antibody was used for detection in ELISA measurements.

As previously reported [7,8], after the breakdown of supersaturation, amyloid fibrils are in equilibrium with monomers. In other words, when the solution reaches equilibrium after amyloid fibril formation, the concentration of the denatured monomers is the same as its solubility, $[D]_C$. Then, we calculated the concentration of the denatured monomers from the concentration of the folded monomers, which were obtained from ELISA measurements, using the equilibrium constant of the folding reaction, $K_F = 4.41\times10^3$. Then, the $[D]_C$ value (= $[N]^E/K_F$) was calculated to be 14.8 pM at 37 °C.

Supplementary Note 9: Temperature dependence of folding and amyloid fibril formation of β2m monomers in the presence of serum albumin

Based on the unified model described in Supplementary Note 10, we here discuss the temperature dependence of folding and amyloid formation of β2m monomers. The calculated denaturation midpoint of β2m monomer increased by ~1 °C (Supplementary Fig. 13b) by the addition of serum albumin at the stoichiometry used in the HANABI assays ([β2m]:[ALB] = 1:0.4), consistent with the experimental results

(Fig. 5h). The presence of serum albumin at the physiological stoichiometries ([β2m]:[ALB] = 1:120–1:4000) would further stabilize the native β2m monomers, increasing the denaturation midpoint by ~2 °C. However, the slight increase in the denaturation midpoint or a fraction of $[D]^S$ ($=[D]^S/([N]^S+[D]^S)$) has a profound effect on the supersaturation ratio (σ = $[D]^S/[D]_C$), an indicator of the risk for the primary nucleation of amyloid fibrils, because $[D]_C$ is a very small value, i.e., 14.8 pM at 37 °C (Supplementary Note 8). The σ value varies by orders of magnitude depending on the status of individuals (Supplementary Fig. 13c). This fact argues that the interactions with serum albumin critically decrease the risk for amyloid fibril formation, even if they change the stability of β2m minimally.

Supplementary Note 10. Unified theoretical model of β2m amyloid fibril formation dependent on β2m and serum albumin concentrations and temperature

(i) Under supersaturation: We first obtained equilibrium concentrations of species under supersaturation where Schemes 1 and 2 in the main body are valid. With the total protein concentrations of serum albumin and β2m as $[\mathrm{ALB}]_\mathrm{T}$ and $[\beta 2\mathrm{m}]_\mathrm{T}$, respectively, $[\mathrm{ALB:N}]^\mathrm{S}$, $[\mathrm{N}]^\mathrm{S}$, and $[\mathrm{D}]^\mathrm{S}$ are represented as functions of $[\mathrm{ALB}]_\mathrm{T}$ and $[\beta 2\mathrm{m}]_\mathrm{T}$:

$$[\mathrm{ALB:N}]^\mathrm{S} = \frac{1}{2K_\mathrm{F}}\left(K_\mathrm{F}[\mathrm{ALB}]_\mathrm{T} + K_\mathrm{F}[\beta 2\mathrm{m}]_\mathrm{T} + (K_\mathrm{F}+1)K_\mathrm{D}^\mathrm{C} - \alpha\right), \quad \text{(S1)}$$

$$[\mathrm{N}]^\mathrm{S} = \frac{1}{2(K_\mathrm{F}+1)}\left(K_\mathrm{F}[\beta 2\mathrm{m}]_\mathrm{T} - K_\mathrm{F}[\mathrm{ALB}]_\mathrm{T} - (K_\mathrm{F}+1)K_\mathrm{D}^\mathrm{C} + \alpha\right), \quad \text{(S2)}$$

$$[\mathrm{D}]^\mathrm{S} = \frac{1}{2K_\mathrm{F}(K_\mathrm{F}+1)}\left(K_\mathrm{F}[\beta 2\mathrm{m}]_\mathrm{T} - K_\mathrm{F}[\mathrm{ALB}]_\mathrm{T} - (K_\mathrm{F}+1)K_\mathrm{D}^\mathrm{C} + \alpha\right), \quad \text{(S3)}$$

where $\alpha = \sqrt{\left(K_\mathrm{F}[\mathrm{ALB}]_\mathrm{T} + K_\mathrm{F}[\beta 2\mathrm{m}]_\mathrm{T} + (K_\mathrm{F}+1)K_\mathrm{D}^\mathrm{C}\right)^2 - 4K_\mathrm{F}^2[\mathrm{ALB}]_\mathrm{T}[\beta 2\mathrm{m}]_\mathrm{T}}$. Here, $K_\mathrm{D}{}^\mathrm{C}$, K_F, and K_P are equilibrium constants for dissociation of serum albumin-native β2m complex, folding/unfolding, and amyloid elongation, respectively, as defined in the main body. It should be noted that concentrations of the three species are linked by equations (1) and (2) in the main body and solving the quadratic equation is required to obtain the concentrations exactly.

(ii) After breakdown of supersaturation: Then, under the conditions where the concentration of denatured monomer exceeds its solubility (i.e., $[\mathrm{D}]^\mathrm{S} > [\mathrm{D}]_\mathrm{C}$), the new equilibrium establishes after breakdown of supersaturation by combining Schemes 1-3 in the main body:

$$[\mathrm{ALB:N}]^\mathrm{E} = \frac{K_\mathrm{F}[\mathrm{D}]_\mathrm{C}}{K_\mathrm{D}^\mathrm{C} + K_\mathrm{F}[\mathrm{D}]_\mathrm{C}}[\mathrm{ALB}]_\mathrm{T}, \quad \text{(S4)}$$

$$[\mathrm{N}]^\mathrm{E} = K_\mathrm{F}[\mathrm{D}]_\mathrm{C}, \quad \text{(S5)}$$

$$[\mathrm{D}]^\mathrm{E} = [\mathrm{D}]_\mathrm{C}, \quad \text{and} \quad \text{(S6)}$$

$$[\mathrm{P}]^\mathrm{E} = [\beta 2\mathrm{m}]_\mathrm{T} - \frac{K_\mathrm{F}[\mathrm{D}]_\mathrm{C}}{K_\mathrm{D}^\mathrm{C} + K_\mathrm{F}[\mathrm{D}]_\mathrm{C}}[\mathrm{ALB}]_\mathrm{T} - (K_\mathrm{F}+1)[\mathrm{D}]_\mathrm{C}. \quad \text{(S7)}$$

Most importantly, the solubility of denatured monomer, $[\mathrm{D}]_\mathrm{C}$, is the dominant parameter determining the overall equilibrium; no consideration of a quadratic equation is necessary.

(iii) Temperature dependence: To include the temperature effects into the unified model, we considered the temperature dependences of three equilibrium constants, $K_\mathrm{D}{}^\mathrm{C}$, K_F, and K_P. Regarding $K_\mathrm{D}{}^\mathrm{C}$, the QCM results (Fig. 5d, e, and Supplementary Fig. 11) showed that $K_\mathrm{D}{}^\mathrm{C}$ remained on the same order of magnitude regardless of the reaction temperature. Then, $K_\mathrm{D}{}^\mathrm{C}$ was assumed to be independent of temperature and was used the value of $K_\mathrm{D}{}^\mathrm{C}$ = 500 μM.

For K_F, we obtained thermodynamic parameters by the fitting analysis for the thermal denaturation curve acquired by means of near-UV CD spectroscopy (Supplementary Fig. 12c). The fitting function is

$$Y = (a_\mathrm{N}T + b_\mathrm{N})\left\{\frac{1}{1+\exp\left(-\frac{\Delta G_\mathrm{F}(T)}{RT}\right)}\right\} + (a_\mathrm{D}T + b_\mathrm{D})\left\{1 - \frac{1}{1+\exp\left(-\frac{\Delta G_\mathrm{F}(T)}{RT}\right)}\right\}, \quad \text{(S8)}$$

$$\Delta G_\mathrm{F}(T) = \Delta H_\mathrm{F}(T_\mathrm{m})\left(1 - \frac{T}{T_\mathrm{m}}\right) + \Delta C_\mathrm{P}^\mathrm{F}(T - T_\mathrm{m}) - T\Delta C_\mathrm{P}^\mathrm{F} \ln\left(\frac{T}{T_\mathrm{m}}\right) . \quad \text{(S9)}$$

Here, a_N , b_N , a_D , and b_D are fitting parameters regarding the change in the background native and denatured states, and $\Delta G_\mathrm{F}(T)$, $\Delta H_\mathrm{F}(T_\mathrm{m})$, T_m, and $\Delta C_\mathrm{P}^\mathrm{F}$ are change in the Gibbs free energy between native and denatured monomer, the enthalpy change at the denaturation midpoint, the temperature of denaturation midpoint, and the change in heat capacity, respectively. The obtained thermodynamic parameters are summarized in Supplementary Table 5, with the literature values from Kardos *et al.* [9], showing reasonable agreement between them. Then, we calculated the temperature dependence of K_F (T) with the obtained parameters:

$$K_\mathrm{F}(T) = \exp\left(-\frac{\Delta G_\mathrm{F}(T)}{RT}\right). \quad \text{(S10)}$$

The calculated $\Delta G_\mathrm{F}(T)$ is shown in Supplementary Fig. 13a.

For K_P, the change in the Gibbs free energy between denatured monomer and amyloid fibril, $\Delta G_\mathrm{P}(T)$, was obtained from solubility of the denatured monomer at T_0 (= 37 °C) (Supplementary Note 8) as

$$\Delta G_\mathrm{P}(T_0) = -RT \ln\left(\frac{1}{[\mathrm{D}]_\mathrm{C}(T_0)}\right) = -64.3\ (\mathrm{kJ/mol}). \quad \text{(S11)}$$

Then, we assumed that the Gibbs free energy equation as used for K_F is valid for K_P [7,9,10]. We used the thermodynamic parameters of the amyloid fibril formation of β2m reported by Ikenoue et al. [10] ($\Delta H_\mathrm{P}(T_0)$ = -73.6 kJ/mol and $\Delta C_\mathrm{P}^\mathrm{P}$ = -5.0 kJ/mol) and following equations to calculate the temperature dependence of $\Delta G_P(T)$,

$$\Delta H_{\mathrm{P}}(T) = \Delta H_{\mathrm{P}}(T_0) + \Delta C_{\mathrm{P}}^{\mathrm{P}}(T - T_0), \quad \text{(S12)}$$

$$\Delta S_{\mathrm{P}}(T) = \Delta S_{\mathrm{P}}(T_0) + \Delta C_{\mathrm{P}}^{\mathrm{P}} \ln\left(\frac{T}{T_0}\right), \text{ and} \quad \text{(S13)}$$

$$\Delta G_{\mathrm{P}}(T) = \Delta H_{\mathrm{P}}(T) - T\Delta S_{\mathrm{P}}(T). \quad \text{(S14)}$$

The calculated $\Delta G_P(T)$ are shown in Supplementary Fig. 13a. The $\Delta G_{\mathrm{P}}(T)$ was converted to $K_{\mathrm{P}}(T)$ by (Eq. S10) as was the conversion of $\Delta G_{\mathrm{F}}(T)$ to $K_{\mathrm{F}}(T)$.

Supplementary Note 11. Estimation of the onset risk of DRA

According to the classical nucleation theory [11], the nucleation rate of a solute in a supersaturated solution, J, is denoted as:

$$J \propto \exp\left(-\frac{\Delta G^{\ddagger}}{RT}\right), \quad \text{(S15)}$$

where $\Delta G^{\ddagger}$, R, and T denote the activation free energy, gas constant, and absolute temperature, respectively. It is known that the $\Delta G^{\ddagger}$ value depends on the supersaturation ratio, σ, written as [11]

$$\Delta G^{\ddagger} \propto \ln^{-2}(\sigma). \quad \text{(S16)}$$

In the amyloid fibril formation, the fibrils are formed by aggregation of the supersaturated denatured monomers. Thus, in this study, the σ value is denoted as:

$$\sigma = \frac{[\mathrm{D}]^{\mathrm{S}}}{[\mathrm{D}]_{\mathrm{C}}}$$
$$= \frac{1}{2[\mathrm{D}]_{\mathrm{C}} K_{\mathrm{F}}(K_{\mathrm{F}}+1)}\left(K_{\mathrm{F}}[\beta 2\mathrm{m}]_{\mathrm{T}} - K_{\mathrm{F}}[\mathrm{ALB}]_{\mathrm{T}} - (K_{\mathrm{F}}+1)K_{\mathrm{D}}^{\mathrm{C}} + \alpha\right), \quad \text{(S17)}$$

where $\alpha = \sqrt{(K_{\mathrm{F}}[\mathrm{ALB}]_{\mathrm{T}} + K_{\mathrm{F}}[\beta 2\mathrm{m}]_{\mathrm{T}} + (K_{\mathrm{F}}+1)K_D^C)^2 - 4K_{\mathrm{F}}^2[\mathrm{ALB}]_{\mathrm{T}}[\beta 2\mathrm{m}]_{\mathrm{T}}}$. Given that the time for nucleation, $t_{\mathrm{nuc.}}$, is inversely proportional to the nucleation rate [12], the following equation is derived:

$$\ln(t_{\mathrm{nuc.}}) \propto \ln(J^{-1}) \propto \ln^{-2}(\sigma). \qquad (\sigma > 1) \qquad \text{(S18)}$$

It should be noted that the nucleation reaction never occurs when $\sigma \leq 1$. Here, we replaced $t_{\mathrm{nuc.}}$ as a lag time for amyloid fibril formation because amyloid fibrils rapidly grow once nuclei form. These relations were used to estimate the onset risk of DRA.

The fluctuation of the temporary risk of amyloid fibril formation with time was calculated based on the change in the total β2m and albumin concentrations with time, as:

$$[\beta2\mathrm{m}]_{\mathrm{T}}(t) = [\beta2\mathrm{m}]_{\mathrm{T}}^{\mathrm{Post}} + \frac{[\beta2\mathrm{m}]_{\mathrm{T}}^{\mathrm{Pre}} - [\beta2\mathrm{m}]_{\mathrm{T}}^{\mathrm{Post}}}{T_{\mathrm{D}}} t, \qquad \text{(S19)}$$

$$[\mathrm{ALB}]_{\mathrm{T}}(t) = [\mathrm{ALB}]_{\mathrm{T}}^{\mathrm{Post}} + \frac{[\mathrm{ALB}]_{\mathrm{T}}^{\mathrm{Post}} - [\mathrm{ALB}]_{\mathrm{T}}^{\mathrm{Pre}}}{T_{\mathrm{D}}} t, \qquad \text{(S20)}$$

where $[\beta2\mathrm{m}]_{\mathrm{T}}(t)$, $[\beta2\mathrm{m}]_{\mathrm{T}}^{\mathrm{Pre}}$, and $[\beta2\mathrm{m}]_{\mathrm{T}}^{\mathrm{Post}}$ are the total concentrations of β2m monomer at time *t* and immediately before and after maintenance dialysis treatment, respectively. The definition of super- and subscripts are the same as for the total concentration of serum albumin. T_{D} is the interval between the maintenance dialysis treatments. By substituting equations (S19) and (S20) into equation (S17), the supersaturation ratio at time *t*, σ(*t*), was calculated, and, by a relation: $TR = \ln^2(\sigma(t))$, the temporary risk of amyloid fibril formation at time *t* was calculated.

Supplementary Figures

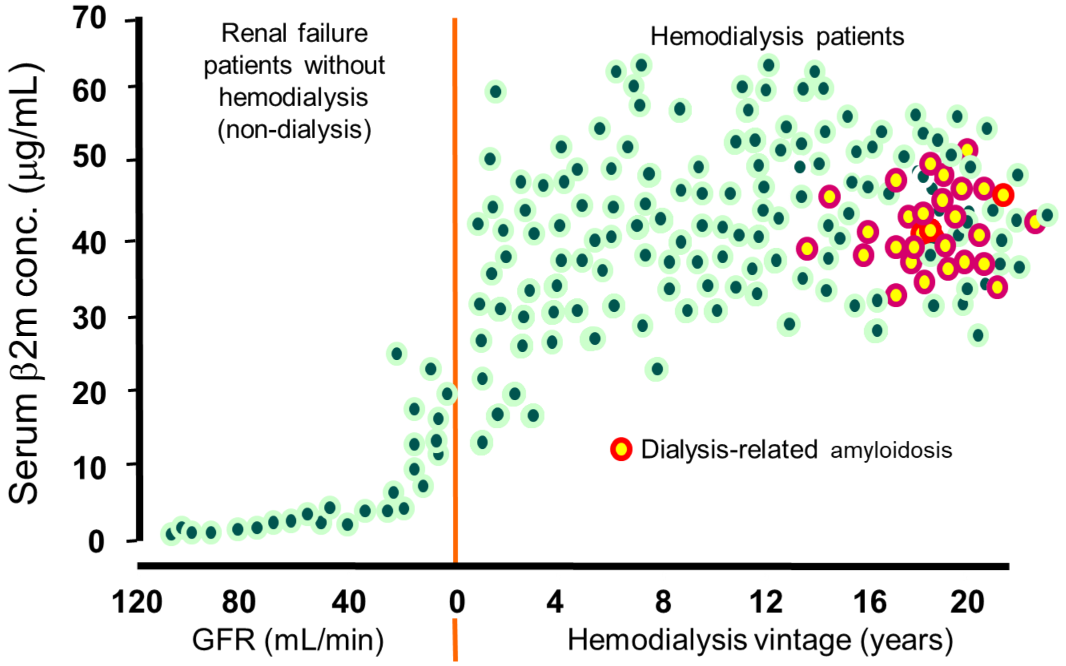

Supplementary Fig. 1. Primary and secondary risk factors for the onset of dialysis-related amyloidosis. Serum β2m concentrations in chronic renal failure patients without hemodialysis vs. with hemodialysis [13, 14]. There is an inverse correlation between the serum β2m concentration and glomerular filtration rate (GFR) in chronic renal failure patients. After the start of hemodialysis, the serum β2m concentration shows a rapid increase and becomes markedly elevated, exceeding the upper limit of normal by 40 times or more. The yellow dots indicate patients with dialysis-related amyloidosis, and the green dots represent patients without amyloidosis. The fact that no differences are apparent in the serum β2m concentrations between dialysis patients with and without amyloidosis indicates that, although the increase in the serum β2m concentration from a healthy level and long dialysis vintage are the primary and secondary risk factors for the onset of DRA, respectively, there are unknown risk factors controlling the onset [14]. This figure is reproduced with permission from Supplementary Reference 14.

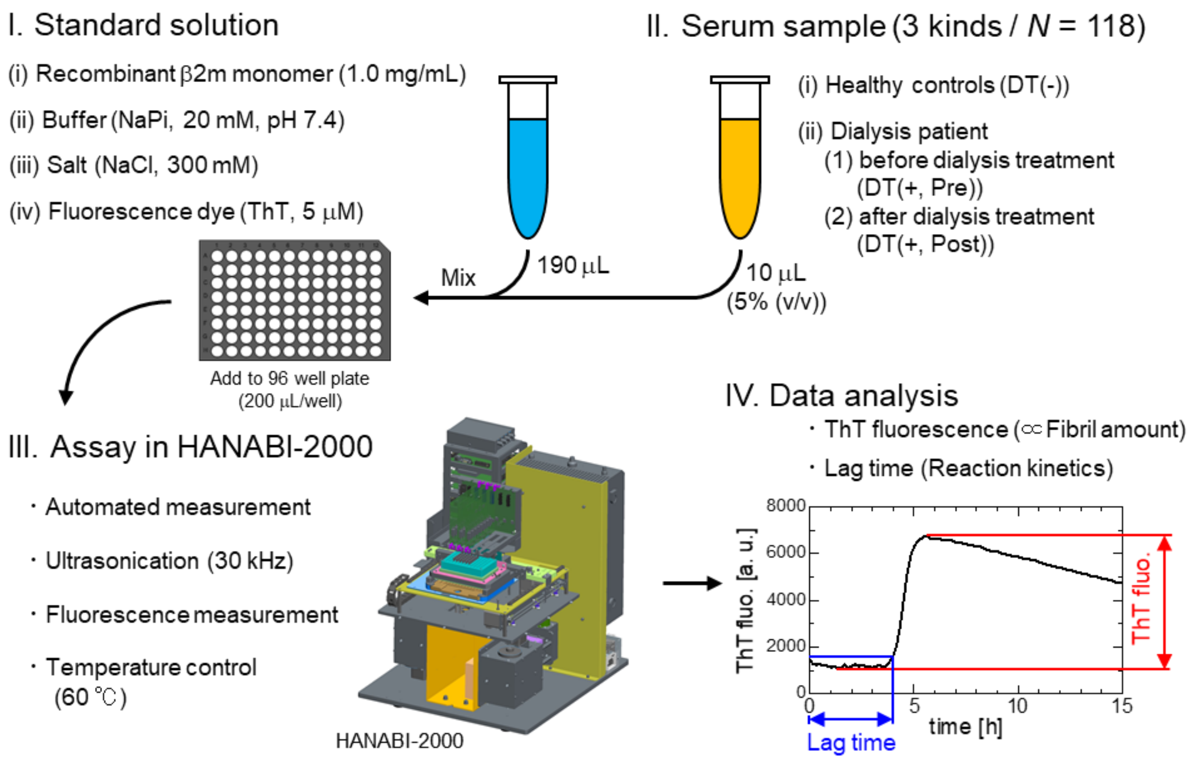

Supplementary Fig. 2. Experimental scheme of the HANABI assay. Schematic illustration of experimental scheme using HANABI-2000 instrument. To examine the effects of sera on the β2m amyloid fibril formation, the sera were added to the standard solution with a volume ratio of 5% (v/v). The aliquots were dispensed in a 96-well plate with the volume of 200 μL/well. The prepared plate was set to the HANABI-2000 instrument and was ultrasonicated at 60 °C. The fibril formation kinetics was monitored by ThT fluorescence measurement. The obtained ThT kinetics was analyzed in terms of its maximum intensity and lag time. The illustration of the HANABI-2000 is reproduced with permission from Supplementary Reference 15.

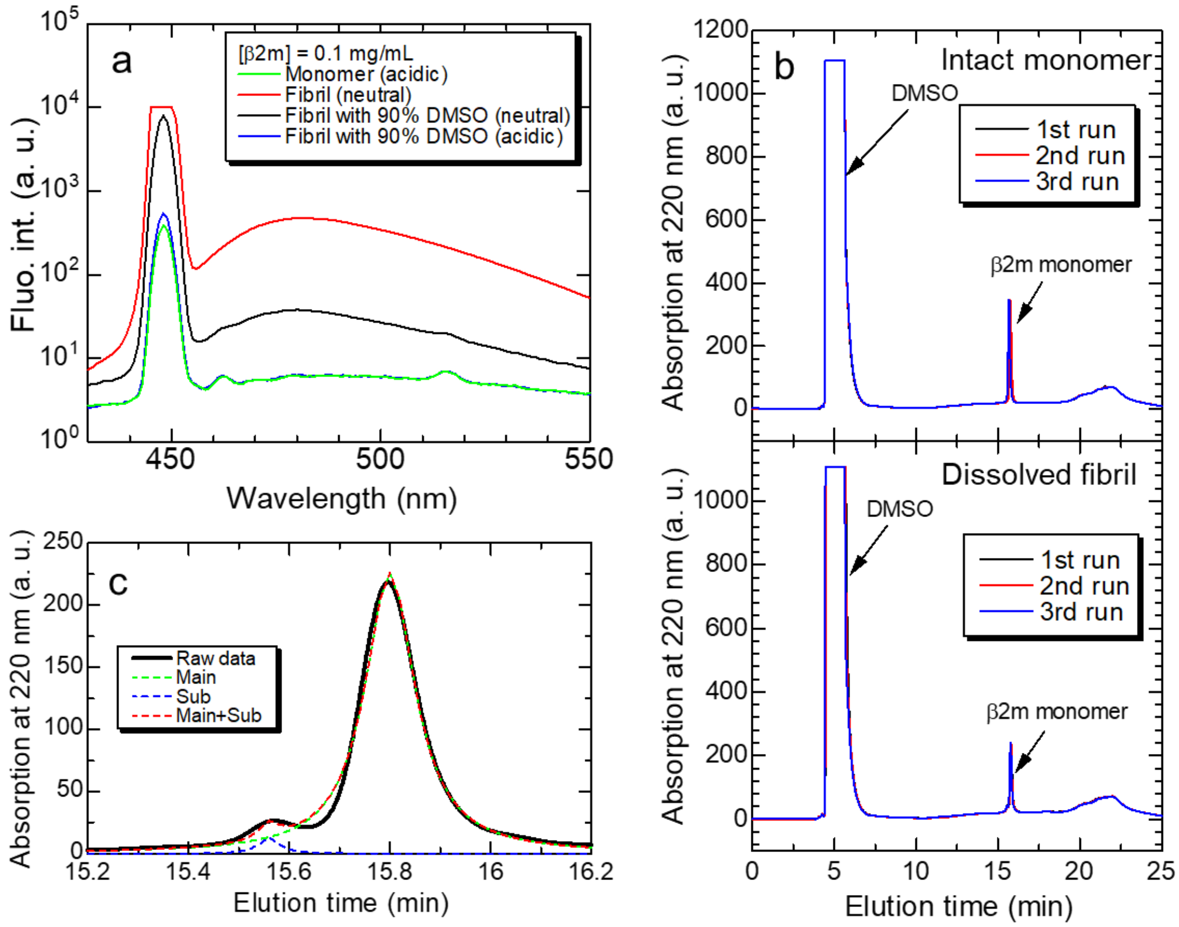

Supplementary Fig. 3. Analysis of amyloid fibrils formed by ultrasonication using reversed-phase chromatography. (a) Amyloid-specific ThT fluorescence spectra of intact monomers and depolymerized amyloid fibrils. Fluorescence measurements were performed with an excitation wavelength of 445 nm. (b) Reversed-phase chromatograms of the intact monomers (upper) and depolymerized amyloid fibrils (lower). The analysis was performed three times for each sample. (c) Enlarged view of the elution peak of the depolymerized amyloid fibrils (solid line) and fitted curves with the Lorentz function (dotted lines). a. u., arbitrary units.

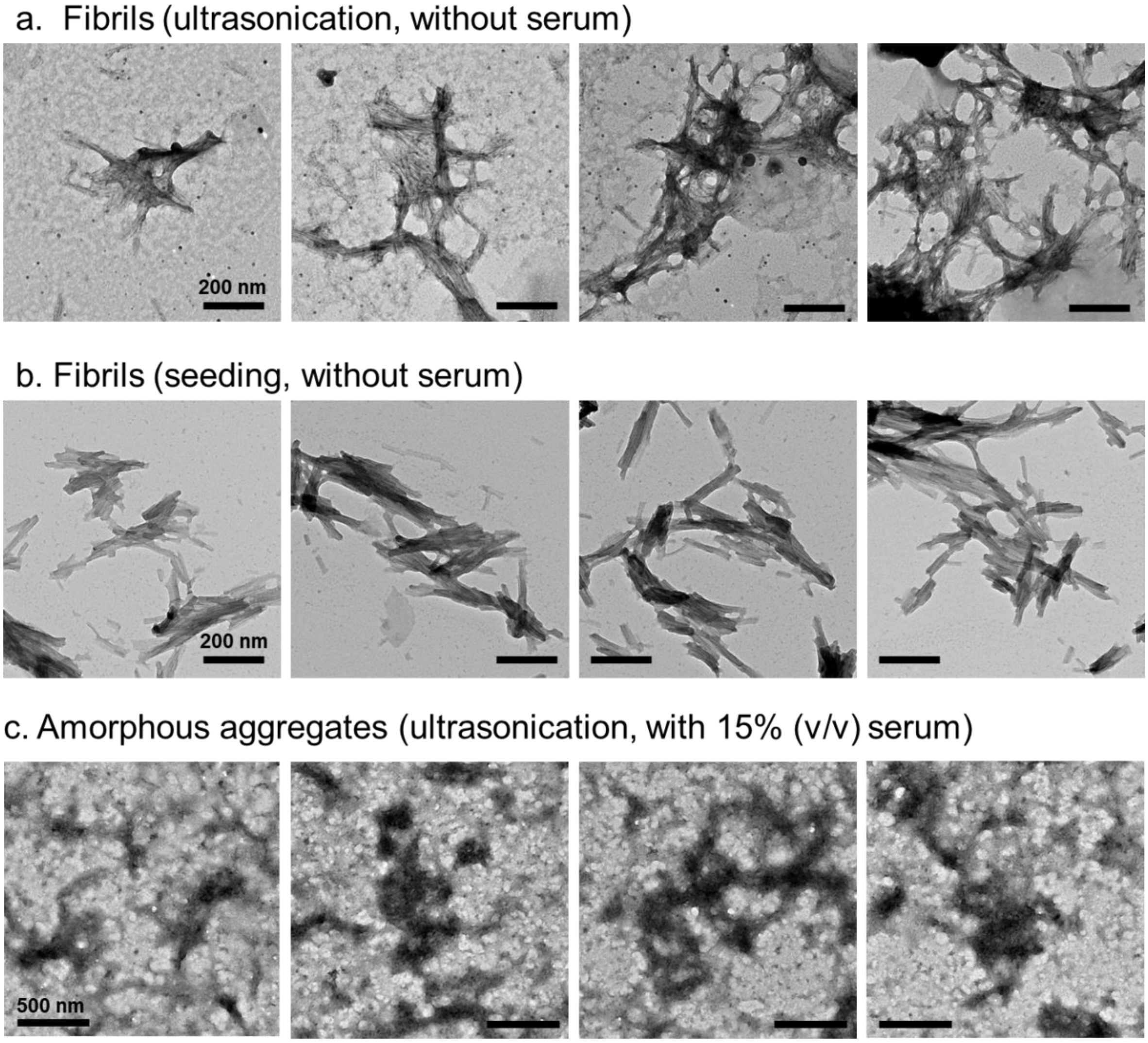

Supplementary Fig. 4. TEM images obtained to confirm reproducibility. Supplementary TEM images of (a) amyloid fibrils formed by ultrasonication without serum addition, (b) amyloid fibrils seeded and elongated from the amyloid fibrils formed by ultrasonication, and (c) amorphous aggregates formed by ultrasonication with 15% (v/v) serum addition. Scale bars in panels a, b, and c denote 200, 200, and 500 nm, respectively.

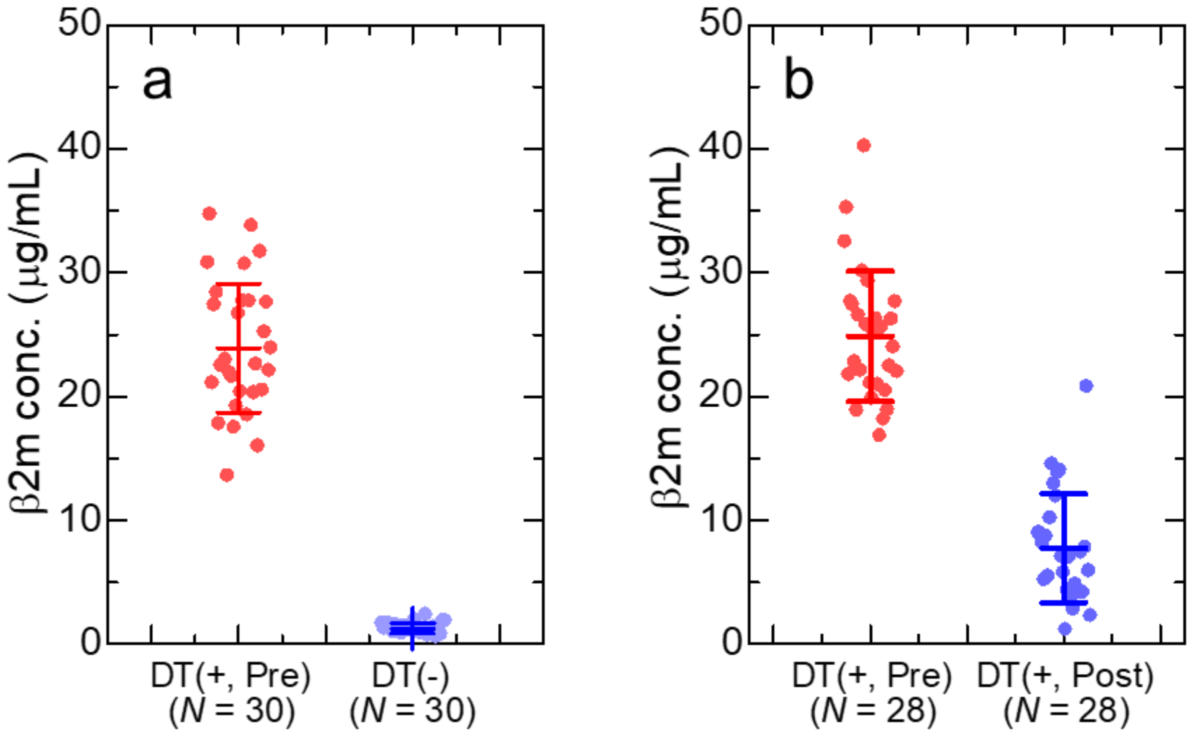

Supplementary Fig. 5. Differences in β2m monomer concentrations in each group.

β2m monomer concentrations in sera used in a series of experiments shown in (a) Fig. 3 (*N* = 30) and (b) Fig. 4 (*N* = 28) in the main body. The sera of DT(+, Pre) in panels a and b were collected from different patient cohorts. The center of error bars and error bars denote the mean and standard deviation among independent serum samples ((a) *N* = 30 and (b) *N* = 28), respectively.

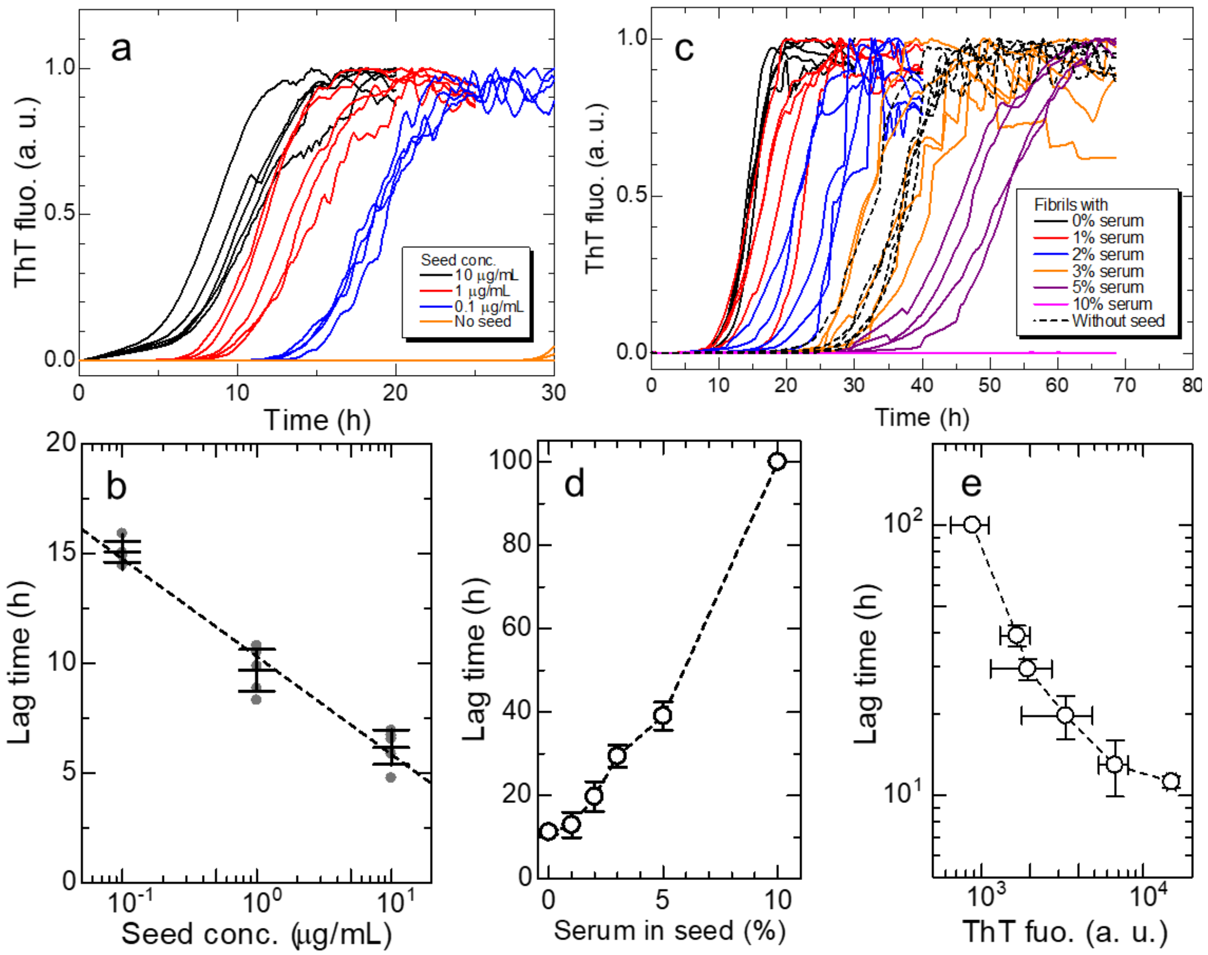

Supplementary Fig. 6. Results of seeding experiments using products at various serum concentrations. (a) ThT fluorescence kinetics of the seeding reaction at various seed concentrations (0.1, 1, and 10 μg/mL) and without seeds. (b) Relationship between the seed concentration and lag time in reactions obtained from the panel a. The error bars denote the standard deviation (n = 5). (c) ThT fluorescence kinetics of the seeding reaction using seeds formed at various serum concentrations from 0-10% (v/v). Relationship between the lag time in the seeding reaction and (d) serum concentration in the seed solution and (e) ThT fluorescence intensity of the seed solution (n = 4). In panels b, d, and e, the centers for error bars and error bars denote the mean and standard deviation. a. u., arbitrary units.

a

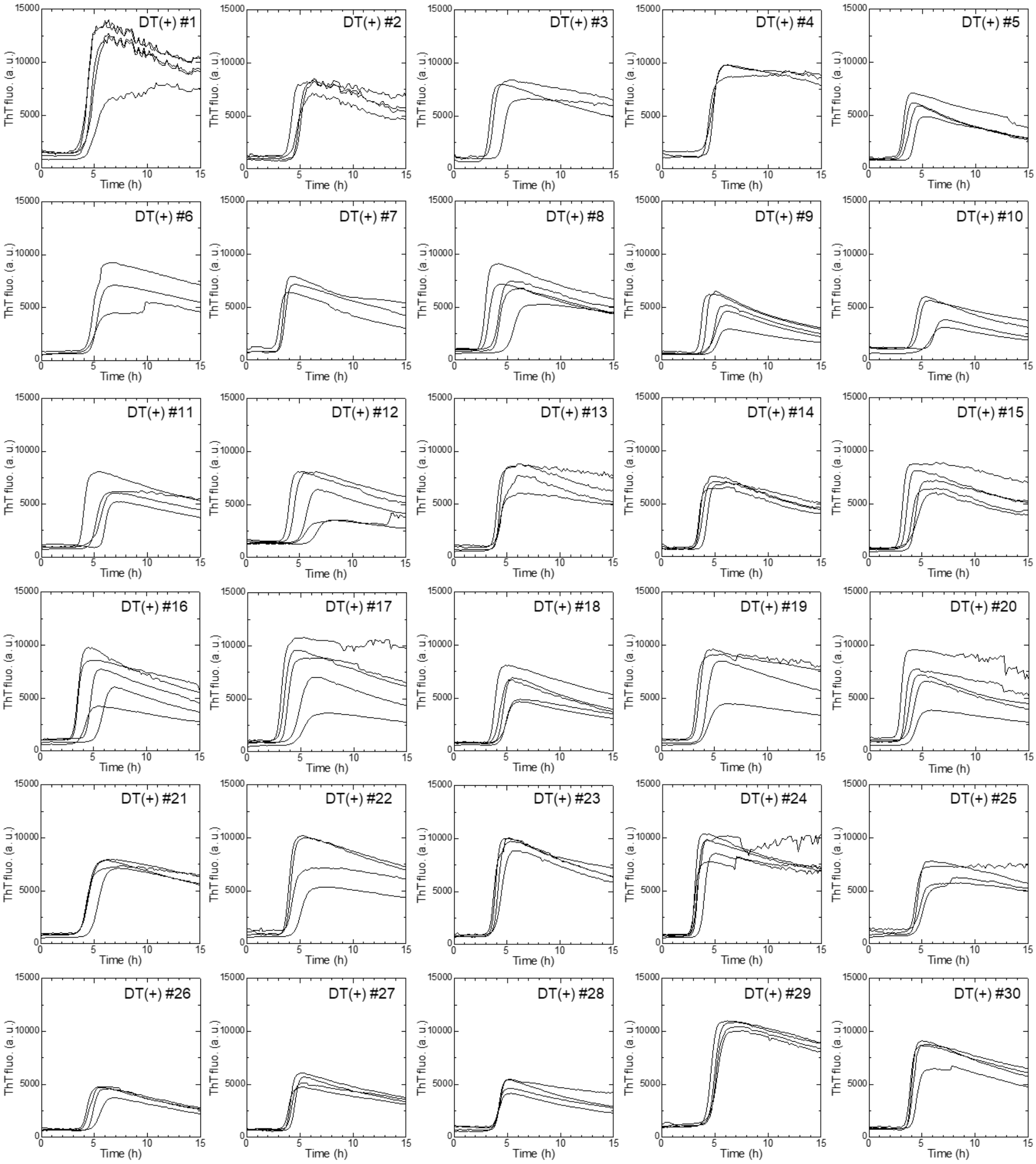

(Continue to the next page)

b

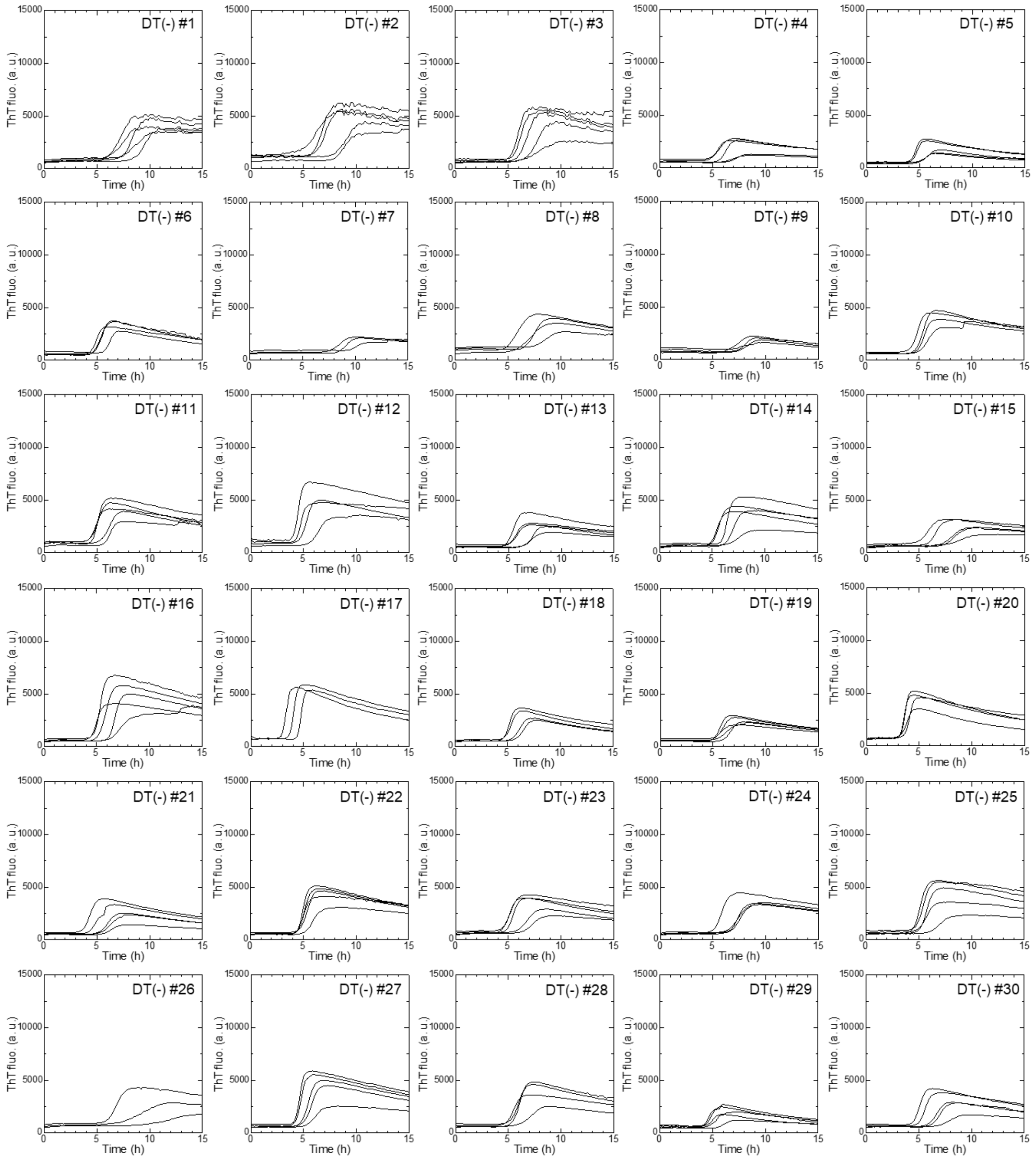

(Continue to the next page)

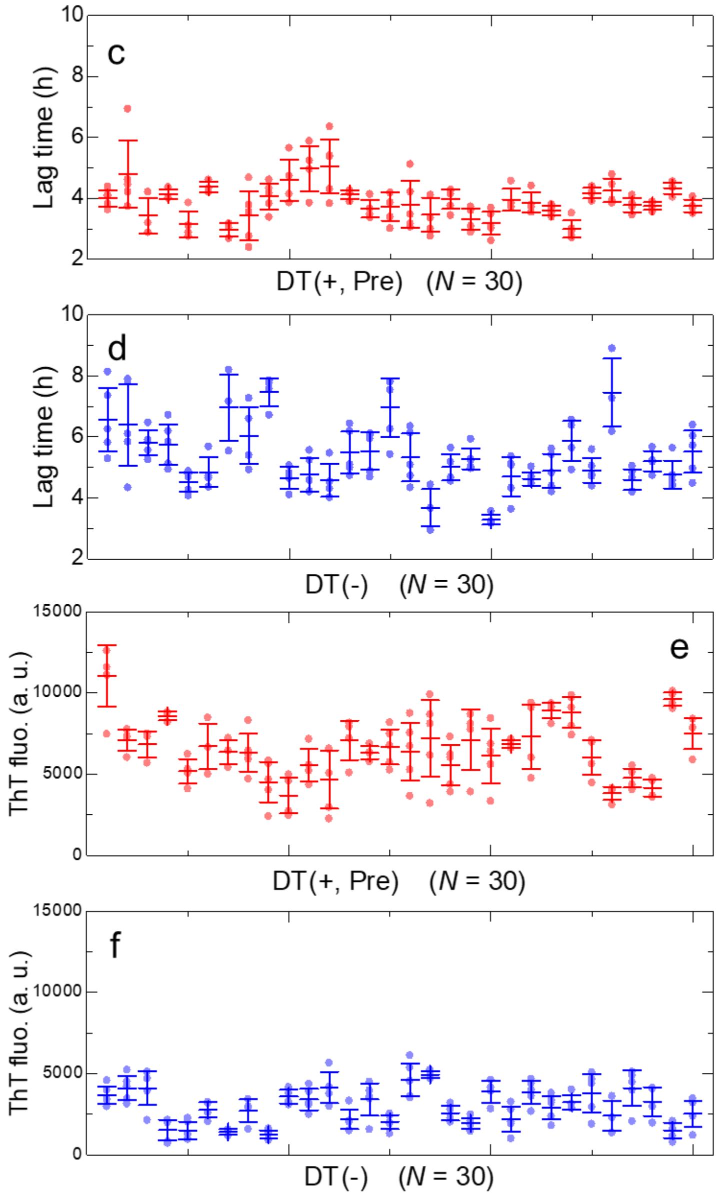

Supplementary Fig. 7. Detailed results of HANABI assay using sera from dialysis patients and non-dialysis controls. ThT kinetics of amyloid fibril formation using (a) sera from dialysis patients (N = 30) and (b) sera from non-dialysis controls (N = 30). For each serum, measurements were performed using at least three independent sample solutions ($n \geq 3$). (c-f) Summary of the ThT fluorescence assay for all samples in terms of the (c,d) lag time and (e,f) ThT fluorescence intensity, respectively. In panels c-f, the centers for error bars and error bars denote the mean and standard deviation, respectively. a. u., arbitrary units.

a

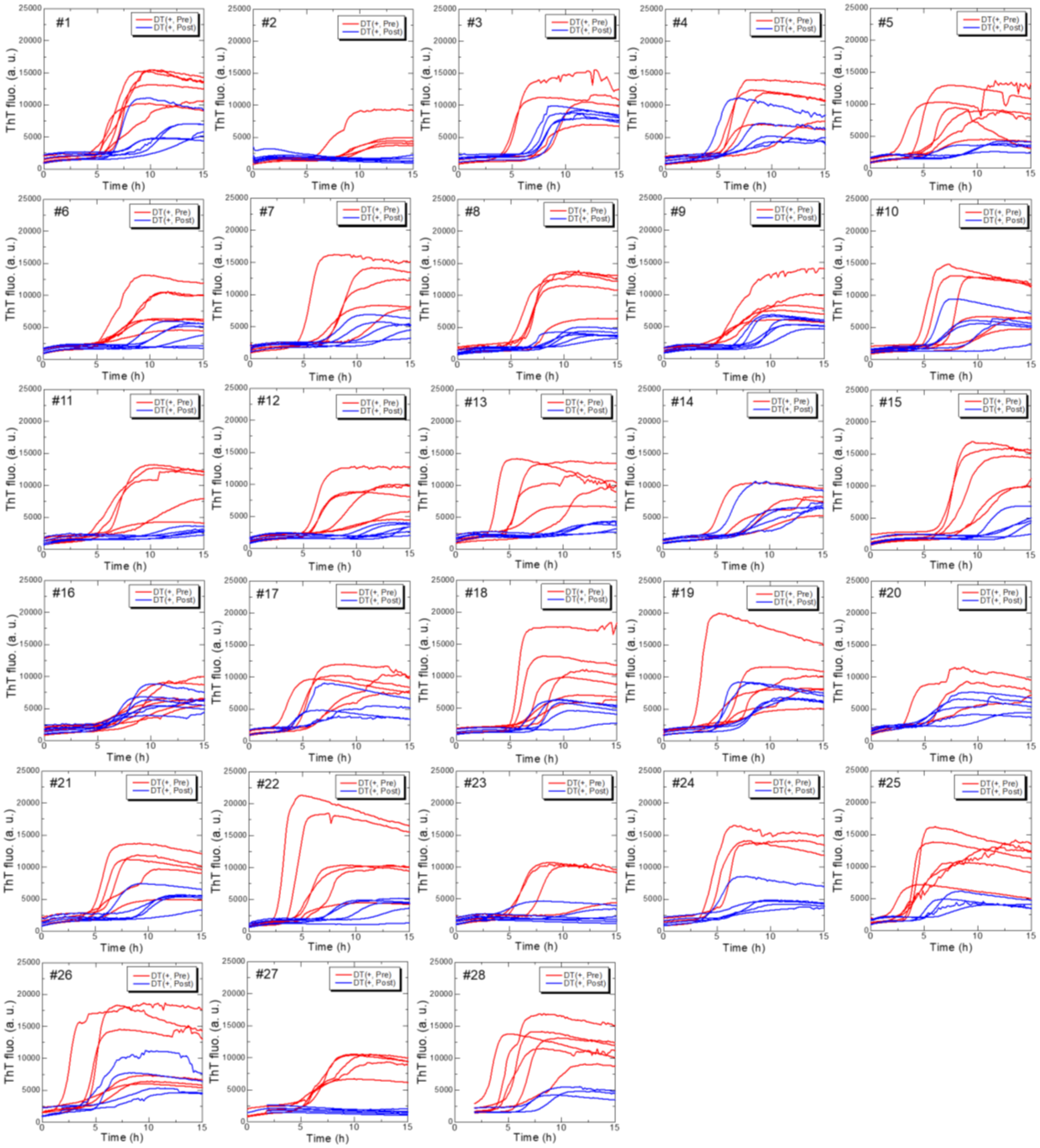

(Continue to the next page)

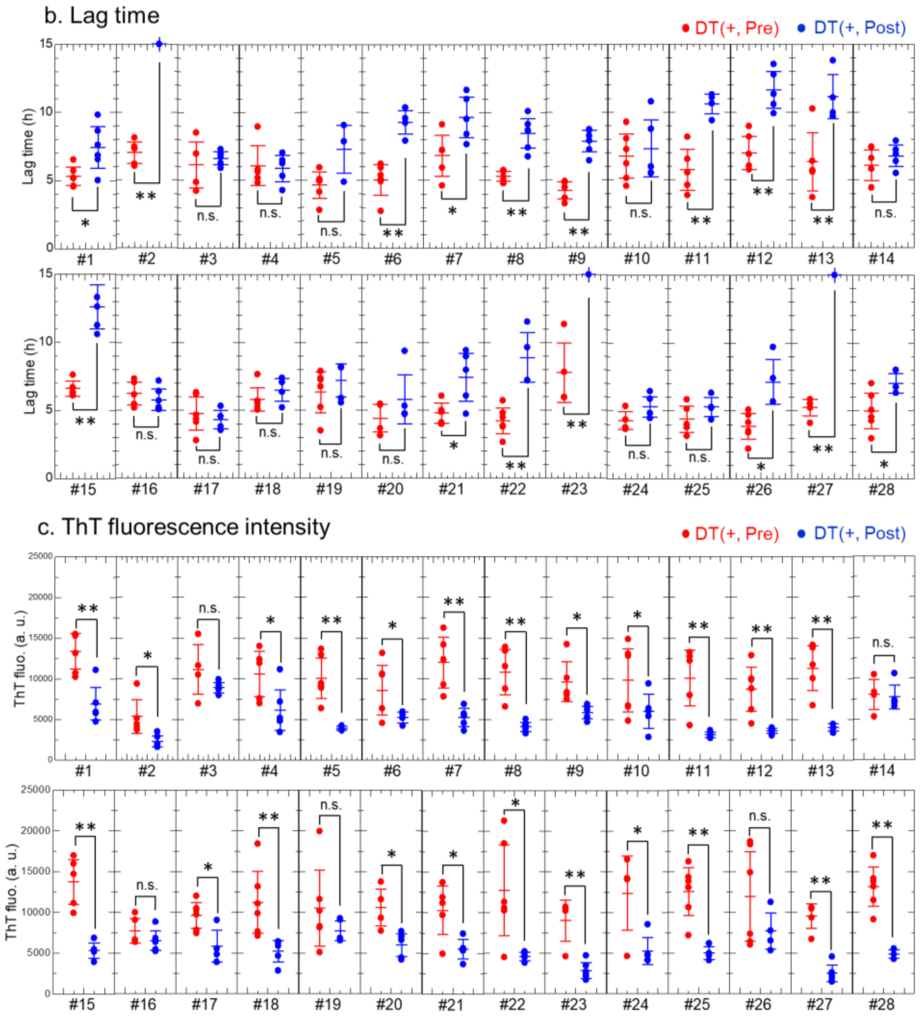

Supplementary Fig. 8. Detailed results of HANABI assay using sera from dialysis patients before and after a single maintenance dialysis. (a) ThT kinetics of amyloid fibril formation using sera from dialysis patients (N = 28) collected immediately before (red curves) and after (blue curves) maintenance dialysis treatments. Test of significance of the (b) lag time and (c) ThT fluorescence intensity of the HANABI assay using sera collected before and after maintenance dialysis treatment ($n \geq 3$). The symbols **, *, and n.s. correspond to the p-values of $p < 0.01$, $0.01 < p < 0.05$, and $p > 0.05$, respectively. The p-value was calculated by the unpaired one-sided t-test, and the exact p-values are provided in Supplementary Table 2. The centers for error bars and error bars denote the mean and standard deviation among independent measurements ($n \geq 3$), respectively. a. u., arbitrary units.

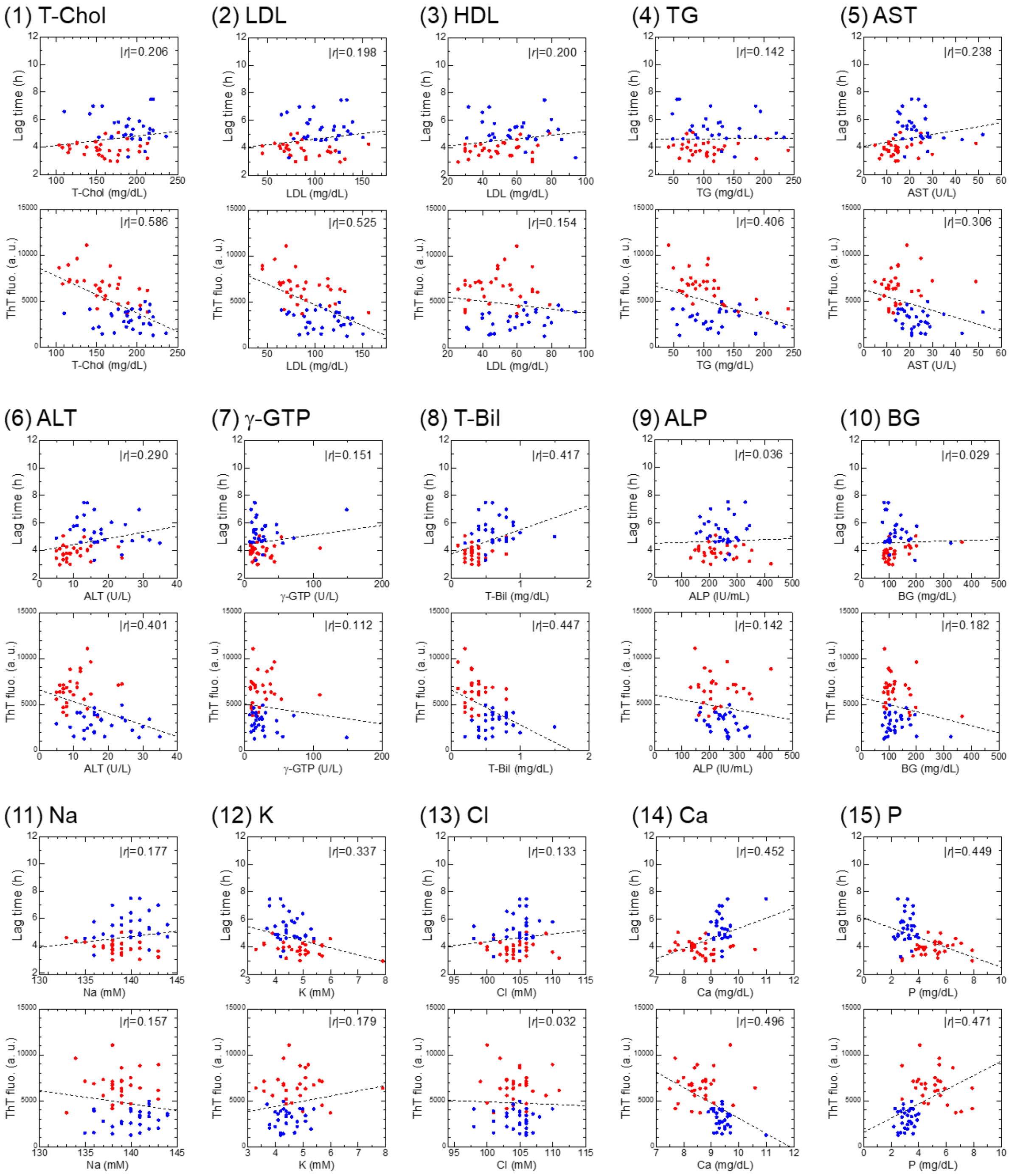

(Continue to the next page)

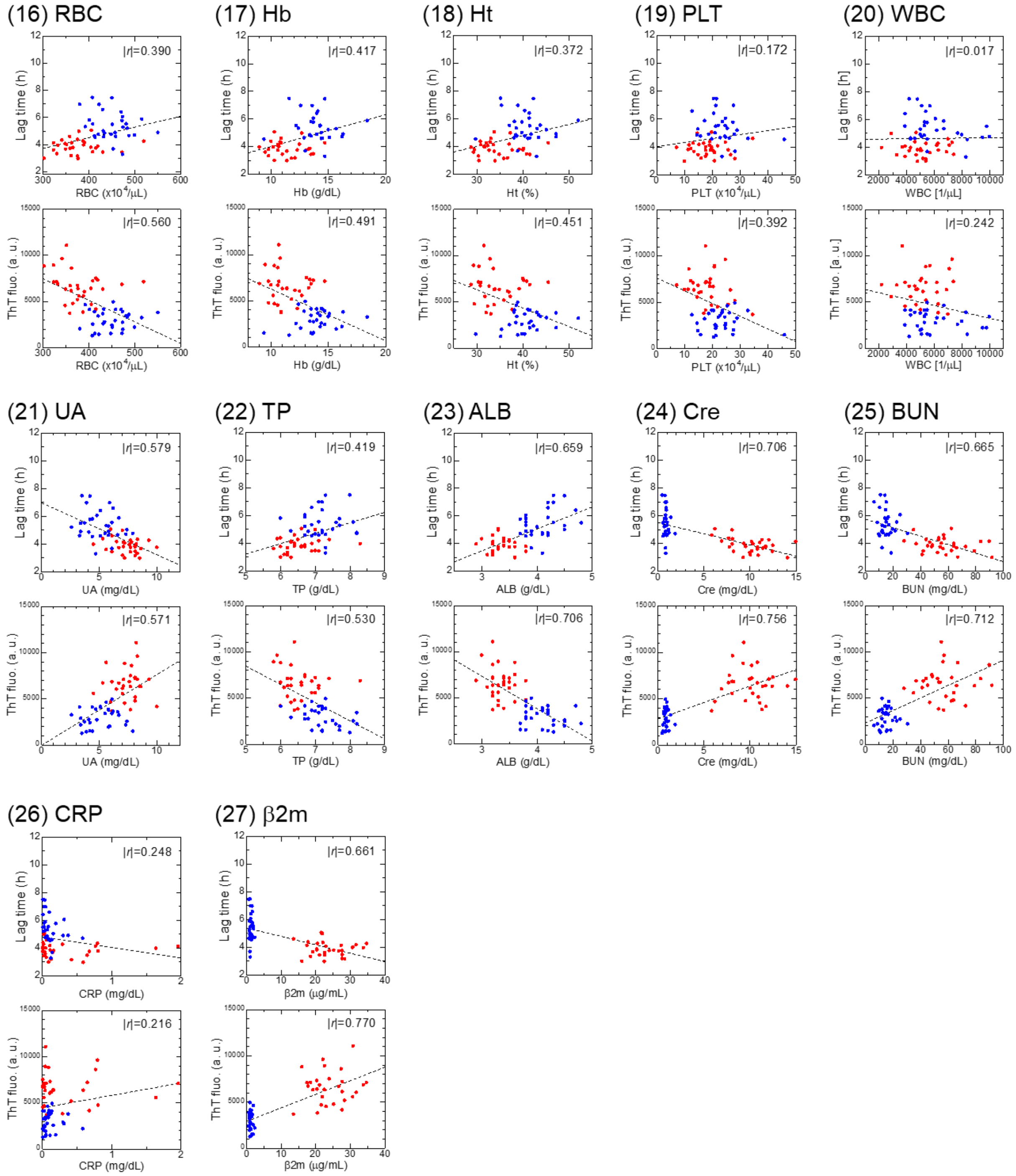

Supplementary Fig. 9. Analysis of correlation coefficients between 27 serum components and results of ThT fluorescence kinetic analysis. For each component, data were fitted by a linear function. Abbreviations of serum components are as follows: (1) T-Chol, total cholesterol; (2) LDL, low-density lipoprotein; (3) HDL, high-density lipoprotein; (4) TG, triglyceride; (5) AST, aspartate acid transaminase; (6) ALT, alanine

aminotransferase; (7) γ-GTP, γ-glutamyl transpeptidase; (8) T-Bil, total bilirubin; (9) ALP, alkaline phosphatase; (10) BG, blood glucose; (16) RBC, red blood cell; (17) Hb, haemoglobin; (18) Ht, hematocrit; (19) PLT, platelet; (20) WBC, white blood cell; (21) UA, ureic acid; (22) TP, total protein; (23) ALB, serum albumin; (24) Cre, creatinine; (25) BUN, blood urea nitrogen; and (26) CRP, C-reactive protein. a. u., arbitrary units.

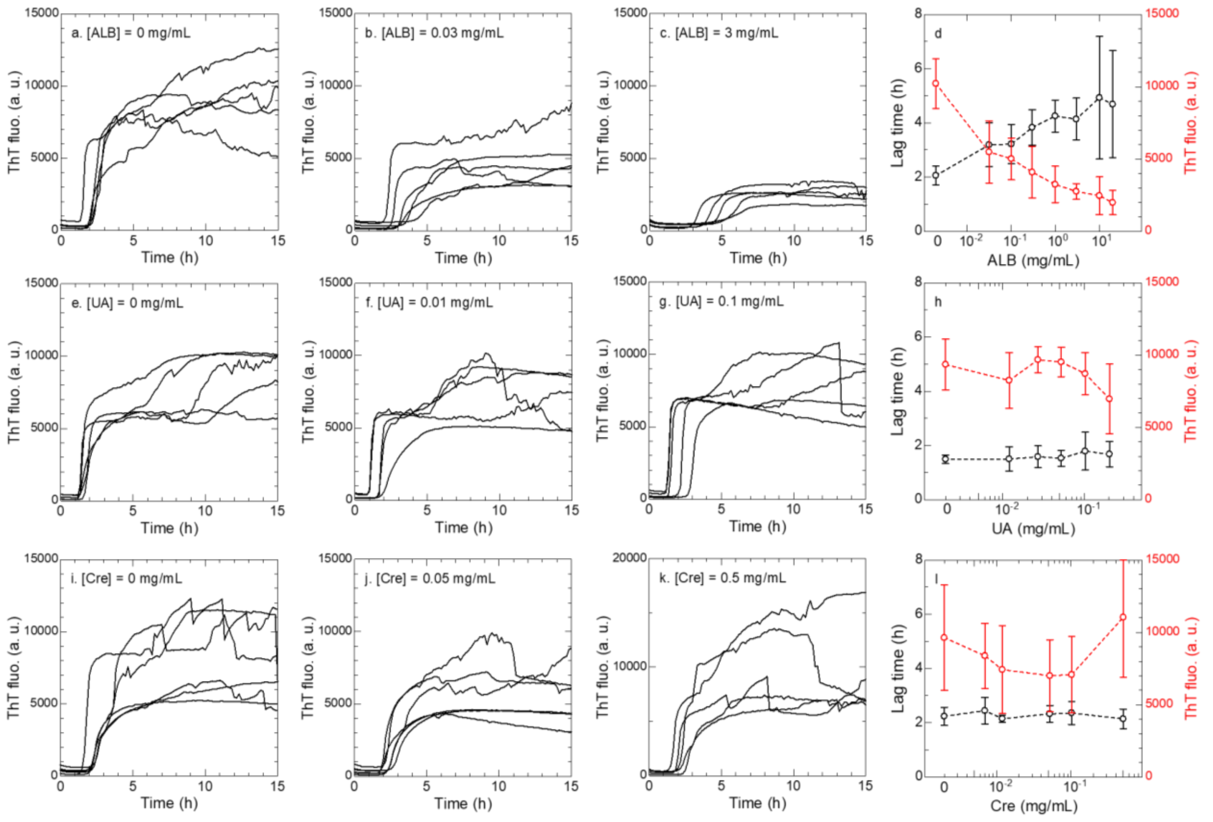

Supplementary Fig. 10. Effects of serum components on β2m amyloid fibril formation. Effects of (a-d) serum albumin (ALB), (e-h) ureic acid (UA), and (i-l) creatinine (Cre) on amyloid fibril formation of recombinant β2m monomers. ThT kinetics of samples including serum albumin with concentrations of (a) 0, (b) 0.03, and (c) 3 mg/mL; ureic acid with concentrations of (e) 0, (f) 0.01, and (g) 0.1 mg/mL; and creatinine with concentrations of (i) 0, (j) 0.05, and (k) 0.5 mg/mL. Dependency of the lag time and ThT fluorescence on (d) serum albumin, (h) ureic acid, and (l) creatinine concentrations are summarized. In panels d, h, and l, the centers for error bars and error bars denote the mean and standard deviation, respectively. For each concentration, measurements were performed using independent sample solutions (n = 5). a. u., arbitrary units.

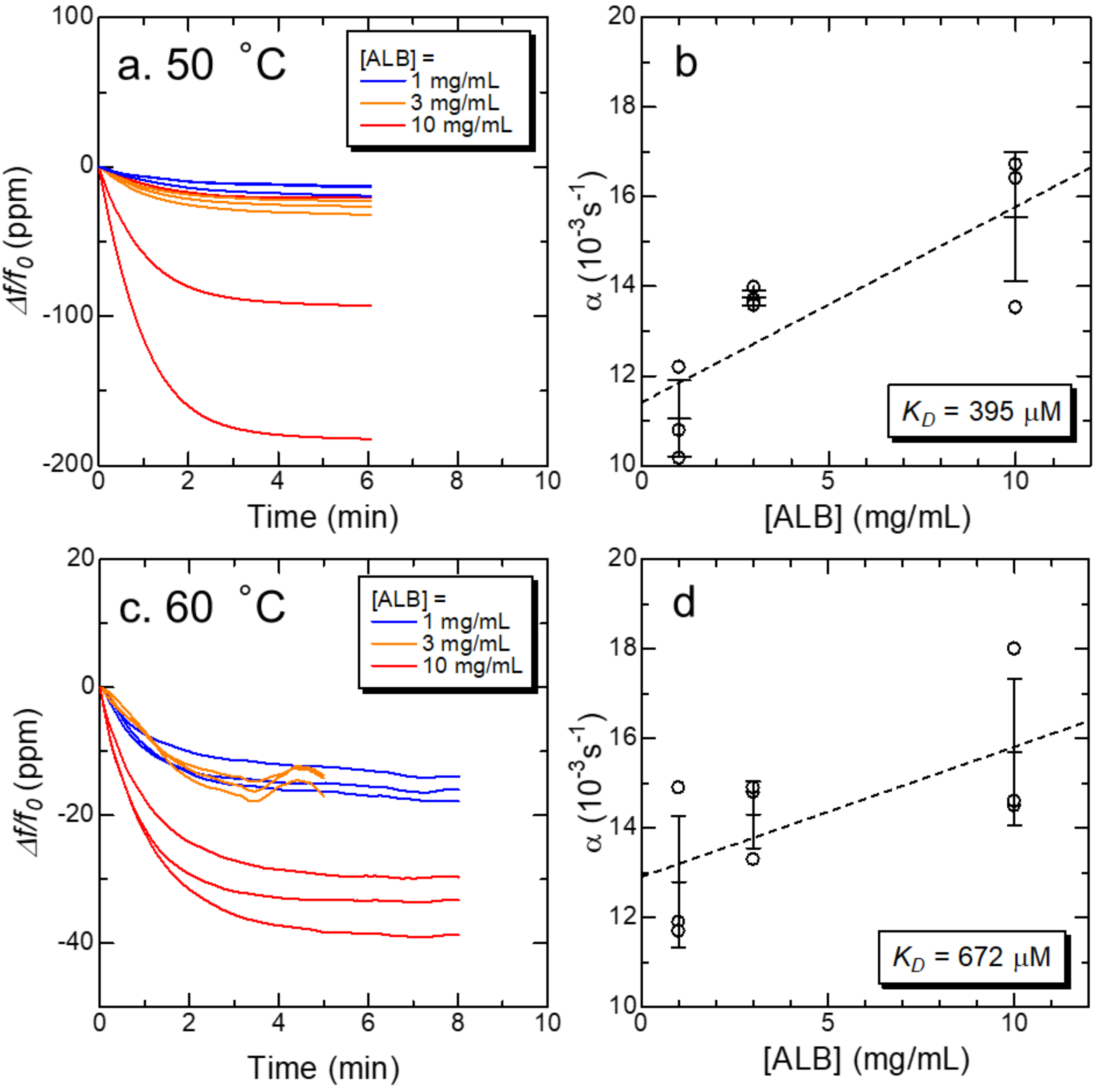

Supplementary Fig. 11. Interactions between β2m monomer and serum albumin at high temperatures monitored by quartz crystal microbalance (QCM). (a, b) 50 and (c, d) 60 °C. (a, c) Resonant frequency change curves of QCM sensors after injection of serum albumin solution with various concentrations at (a) 50 and (c) 60 °C. The measurements were performed using independent sensor chips (n = 3). (b, d) Plots of the α value against serum albumin concentration at (b) 50 and (d) 60 °C, respectively (n = 3). K_D values show the dissociation constant at each temperature. The error bars denote the standard deviation among independent measurements (n = 3).

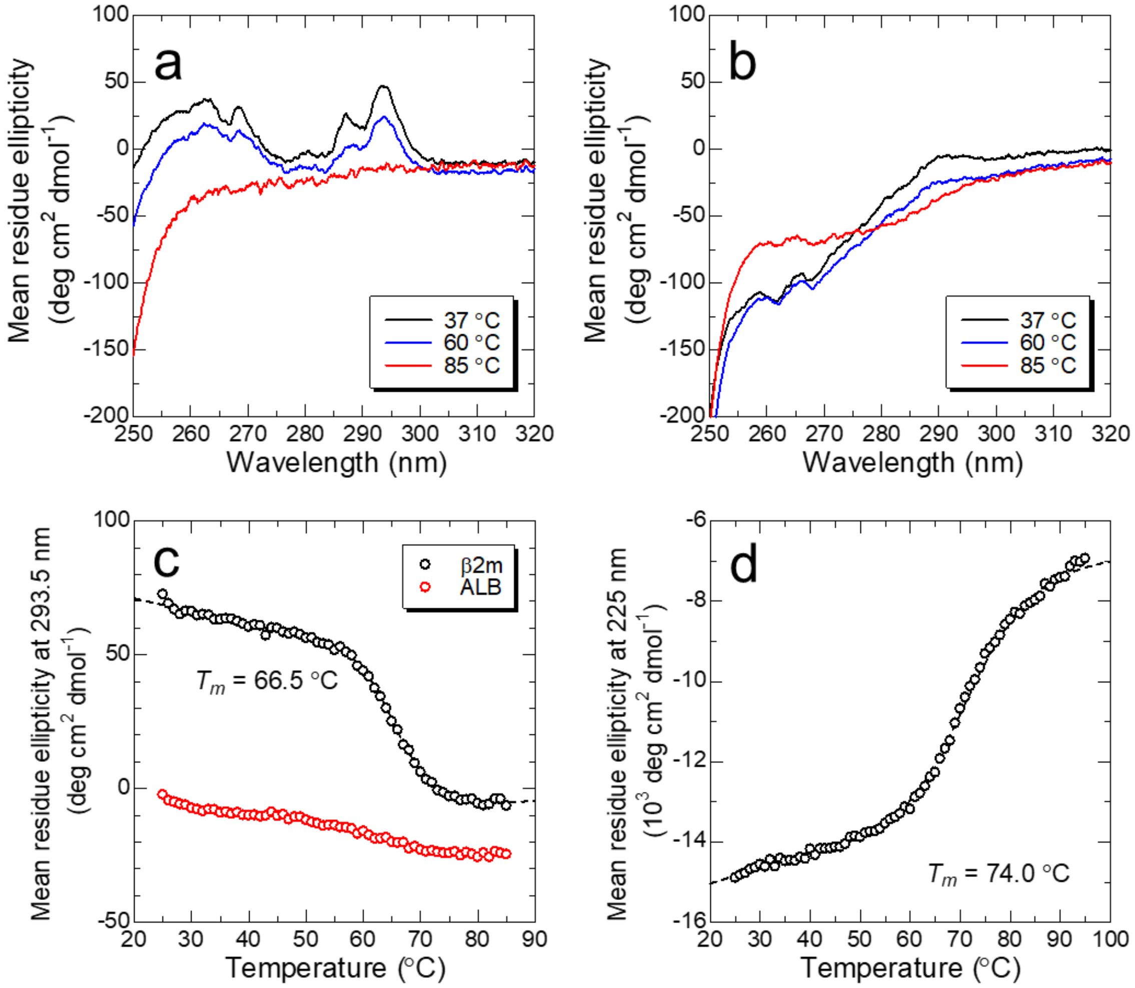

Supplementary Fig. 12. Thermal denaturation of β2m and serum albumin monitored by near-UV CD. Near-UV CD spectra of (a) β2m monomer and (b) serum albumin at various temperatures. (c) Thermal denaturation curves of β2m monomer and serum albumin monitored at 293.5 nm. (d) Thermal denaturation curve of serum albumin monitored at 225 nm, indicating a cooperative denaturation with the midpoint of 74.0 °C.

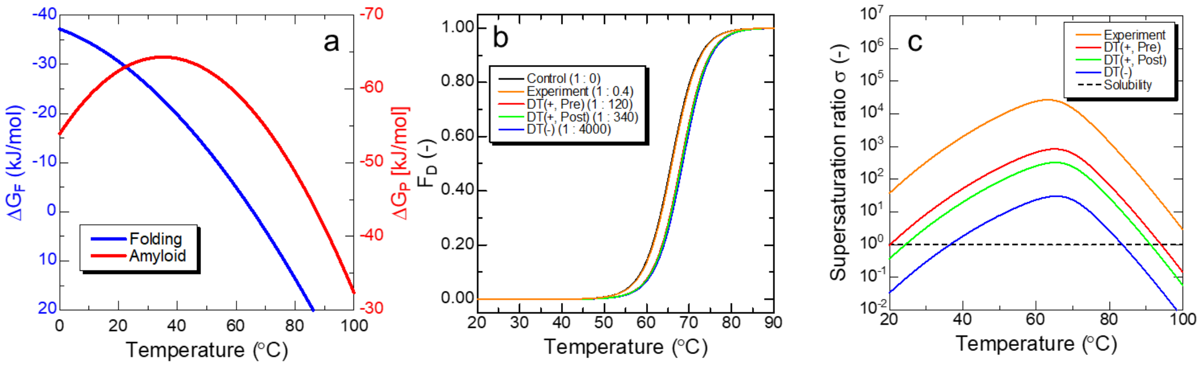

Supplementary Fig. 13. Temperature dependent folding reaction and amyloid fibril formation of β2m monomers in the presence of serum albumin. (a) Temperature dependences of the Gibbs free energy changes of folding, $\Delta G_{\mathrm{F}}(T)$, and amyloid formation, $\Delta G_{\mathrm{P}}(T)$, of denatured monomers. (b) Temperature dependences of F_D, where F_D = [D]/([C]+[N]+[D]), at varying stoichiometries between β2m monomer and serum albumin. The calculation was performed with the representative stoichiometries in the experiments; dialysis patients before (DT(+),Pre) and after (DT(+), Post) maintenance dialysis, and non-dialysis controls (DT(-)). The values in the bracket indicates the stoichiometry of [β2m] : [ALB]. (c) Temperature dependences of the supersaturation ratio, σ, calculated under following conditions; Experiment, [β2m] = 1.0 mg/mL and [ALB] = 2.25 mg/mL; DT(+, Pre), [β2m] = 0.05 mg/mL and [ALB] = 35 mg/mL; DT(+, Post), [β2m] = 0.02 mg/mL and [ALB] = 38 mg/mL; and DT(-), [β2m] = 0.002 mg/mL and [ALB] = 45 mg/mL, which are representative values in the HANABI assays, dialysis patients before and after maintenance dialysis treatments, and non-dialysis controls, respectively.

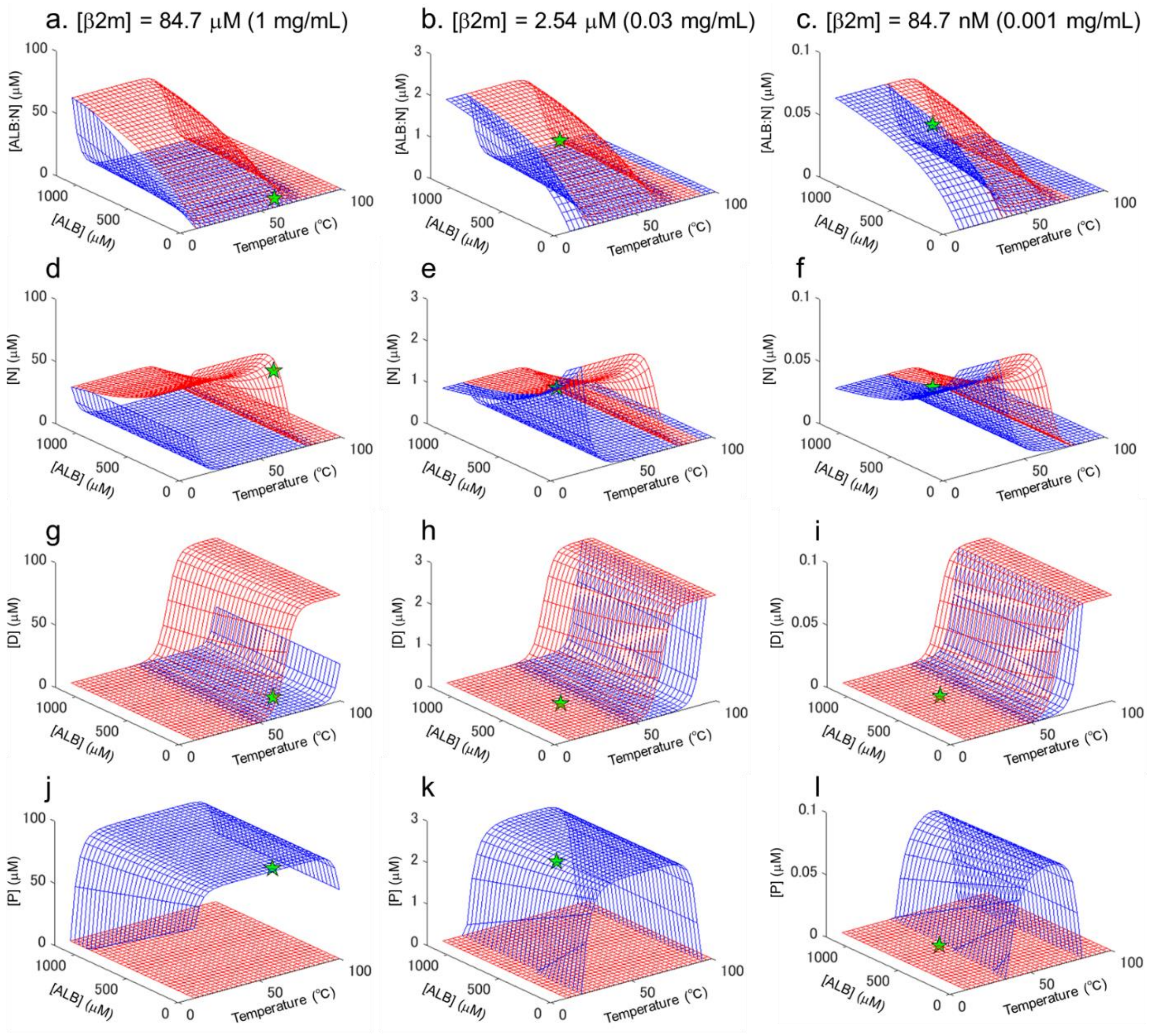

Supplementary Fig. 14. Temperature and serum albumin concentration dependencies of β2m species. The concentrations of (a-c) native β2m-serum albumin complex, (d-f) native and (g-i) denatured β2m monomers, and (j-l) amyloid fibrils under various total β2m concentrations. The calculation was performed with total β2m concentrations of (a,d,g,j) 84.7 μM (1.0 mg/mL), (b,e,h,k) 2.54 μM (0.03 mg/mL), and (c,f,i,l) 84.7 nM (0.001 mg/mL), which correspond to representative β2m monomer concentrations in the HANABI assays, dialysis patients, and non-dialysis controls, respectively. The red and blue mesh surfaces indicate the concentration before and after breakdown of supersaturation, respectively.

Supplementary Table 1. Summary of the examination of significance shown in Supplementary Figure 8b, c.

	Lag time	ThT fluo.
$p > 0.05$ (n.s.)	12	5
$0.01 < p < 0.05$ (*)	5	10
$p < 0.01$ (**)	11	13

Supplementary Table 2. The *p*-values in Figure S8b, c.

	Lag time	ThT fluo.
#1	1.29×10^{-2}	3.54×10^{-4}
#2	1.80×10^{-5}	1.83×10^{-2}
#3	3.34×10^{-1}	1.51×10^{-1}
#4	4.11×10^{-1}	1.87×10^{-2}
#5	7.05×10^{-2}	1.42×10^{-3}
#6	2.94×10^{-4}	2.74×10^{-2}
#7	1.45×10^{-2}	4.64×10^{-3}
#8	4.79×10^{-4}	4.43×10^{-4}
#9	1.86×10^{-5}	1.54×10^{-2}
#10	3.37×10^{-1}	4.93×10^{-2}
#11	6.87×10^{-4}	7.94×10^{-3}
#12	3.28×10^{-4}	4.34×10^{-3}
#13	1.00×10^{-3}	3.13×10^{-3}
#14	1.99×10^{-1}	4.15×10^{-1}
#15	4.62×10^{-4}	1.07×10^{-3}
#16	2.10×10^{-1}	1.20×10^{-1}
#17	2.66×10^{-1}	1.76×10^{-2}
#18	1.49×10^{-1}	8.52×10^{-3}
#19	9.48×10^{-2}	1.20×10^{-1}
#20	1.24×10^{-1}	1.36×10^{-2}
#21	2.04×10^{-2}	1.54×10^{-2}
#22	7.17×10^{-3}	1.15×10^{-2}
#23	5.35×10^{-3}	8.05×10^{-3}
#24	5.24×10^{-2}	3.12×10^{-2}
#25	9.74×10^{-2}	7.78×10^{-4}
#26	1.74×10^{-2}	8.53×10^{-2}
#27	1.63×10^{-6}	4.08×10^{-5}
#28	1.93×10^{-2}	1.78×10^{-4}

Supplementary Table 3. Summary of correlation-coefficient analysis for identical serum components for the lag time.

Rank	Serum component	Correlation-coefficient
1	Cre	0.706
2	BUN	0.665
3	β2m	0.661
4	ALB	0.659
5	UA	0.579
6	Ca	0.452
7	P	0.449
8	TP	0.419
9	Hb	0.417
10	T-Bil	0.417

Supplementary Table 4. Summary of correlation-coefficient analysis for identical serum components for the ThT fluorescence

Rank	Serum component	Correlation-coefficient
1	β2m	0.770
2	Cre	0.756
3	BUN	0.712
4	ALB	0.706
5	T-Chol	0.586
6	UA	0.571
7	RBC	0.560
8	TP	0.530
9	LDL	0.525
10	Ca	0.496

Supplementary Table 5. Thermodynamic parameters of folding reaction of β2m monomer at 37 °C

	ΔH_F (kJ/mol)	$-T\Delta S_F$ (kJ/mol)	ΔG_F (kJ/mol)	ΔC_P^F (kJ/mol)
This study	-211.7	190.2	-21.5	-3.1
Kardos et al. [9]	-174.7	155.6	-19.1	-5.0

Supplementary References

1 Hoshino, J. *et al.* Carpal tunnel surgery as proxy for dialysis-related amyloidosis: results from the Japanese society for dialysis therapy. *Am J Nephrol* **39**, 449-458 (2014).

2 Hoshino, J. *et al.* Significance of the decreased risk of dialysis-related amyloidosis now proven by results from Japanese nationwide surveys in 1998 and 2010. *Nephrol Dial Transplant* **31**, 595-602 (2016).

3 Jarrett, J. T. & Lansbury, P. T., Jr. Seeding "one-dimensional crystallization" of amyloid: a pathogenic mechanism in Alzheimer's disease and scrapie? *Cell* **73**, 1055-1058 (1993).

4 Shahnawaz, M. *et al.* Development of a biochemical diagnosis of Parkinson disease by detection of α-synuclein misfolded aggregates in cerebrospinal fluid. *JAMA Neurol* **74**, 163-172 (2017).

5 Das, A. & Mukhopadhyay, C. Urea-mediated protein denaturation: a consensus view. *J Phys Chem B* **113**, 12816-12824 (2009).

6 Zhang, C. M. *et al.* Possible mechanisms of polyphosphate-induced amyloid fibril formation of β2-microglobulin. *Proc Natl Acad Sci U S A* **116**, 12833-12838 (2019).

7 Noji, M. *et al.* Heating during agitation of β2-microglobulin reveals that supersaturation breakdown is required for amyloid fibril formation at neutral pH. *J Biol Chem* **294**, 15826-15835 (2019).

8 Noji, M. *et al.* Breakdown of supersaturation barrier links protein folding to amyloid formation. *Commun Biol* **4**, 120 (2021).

9 Kardos, J., Yamamoto, K., Hasegawa, K., Naiki, H. & Goto, Y. Direct measurement of the thermodynamic parameters of amyloid formation by isothermal titration calorimetry. *J Biol Chem* **279**, 55308-55314 (2004).

10 Ikenoue, T. *et al.* Heat of supersaturation-limited amyloid burst directly monitored by isothermal titration calorimetry. *Proc Natl Acad Sci USA* **111**, 6654-6659 (2014).

11 Mullin, J. W. *Crystallization*. 4th ed edn, (2001).

12 Guo, Z., Jones, A. G. & Li, N. The effect of ultrasound on the homogeneous nucleation of $BaSO_4$ during reactive crystallization. *Chem Eng Sci* **61**, 1617-1626 (2006).

13 Gejyo, F., Homma, N., Suzuki, Y. & Arakawa, M. Serum levels of β2-microglobulin as a new form of amyloid protein in patients undergoing long-term hemodialysis. *N Engl J Med* **314**, 585-586 (1986).

14. Gejyo, F., (2019) Dialysis-related amyloidosis -from the beginning of the research to the future- In Retire. Fumitake Gejyo from Pres. Niigata Univ.

15. Nakajima, K. *et al.* Optimized sonoreactor for accelerative amyloid-fibril assays through enhancement of primary nucleation and fragmentation. *Ultrason Sonochem* **73**, 105508 (2021).
