# Supplementary material for: Macromolecular crowding and supersaturation protect hemodialysis patients from the onset of dialysis-related amyloidosis
Source: Nat Commun. 2022 Oct 3;13:5689. doi: 10.1038/s41467-022-33247-3 (PMC9530240; doi:10.1038/s41467-022-33247-3)
Supplement: Supplementary file 3 — Description of Additional Supplementary Files [file 41467_2022_33247_MOESM3_ESM.pdf]

Description of Additional Supplementary Files

Supplementary Movie 1- (i) TR under supersaturation: β2m species are in apparent equilibrium between three states, serum albumin-native monomer complex, native monomer, and denatured monomer due to the barrier of supersaturation. TR values vary with the total concentration of β2m and serum albumin. Maintenance dialysis treatment temporarily alleviates TR values by lowering the β2m concentration and increasing the serum albumin concentration. (ii) AR during long dialysis vintage: The risk for the onset of DRA accumulates during a long dialysis vintage. (iii) Breakdown of supersaturation and onset of DRA: When the AR value reaches a threshold, supersaturation is broken, and then, amyloid fibril formation begins. When [D] becomes [D]c, amyloid formation finishes, and the entire solution is truly in equilibrium

Supplementary Data 1- (a) Demographic and clinical characteristics of the non-dialysis controls (N = 30) and dialysis patients (N = 30). (b) Summary of clinical data for the non-dialysis control and dialysis patient groups. (c) Demographic and clinical characteristics of the dialysis patients (N = 28) who were collected before and after a single maintenance dialysis treatment. (d) Summary of clinical data for the dialysis patients in the sheet c.
