# Supplementary material for: Macromolecular crowding and supersaturation protect hemodialysis patients from the onset of dialysis-related amyloidosis
Source: Nat Commun. 2022 Oct 3;13:5689. doi: 10.1038/s41467-022-33247-3 (PMC9530240; doi:10.1038/s41467-022-33247-3)
Supplement: Supplementary file 6 — Reporting Summary [file 41467_2022_33247_MOESM6_ESM.pdf]

nature portfolio

Corresponding author(s): Yuji Goto

Last updated by author(s): Aug 23, 2022

Reporting Summary

Nature Portfolio wishes to improve the reproducibility of the work that we publish. This form provides structure for consistency and transparency in reporting. For further information on Nature Portfolio policies, see our Editorial Policies and the Editorial Policy Checklist.

Statistics

For all statistical analyses, confirm that the following items are present in the figure legend, table legend, main text, or Methods section.

n/a	Confirmed	
☐	☒	The exact sample size (*n*) for each experimental group/condition, given as a discrete number and unit of measurement
☐	☒	A statement on whether measurements were taken from distinct samples or whether the same sample was measured repeatedly
☐	☒	The statistical test(s) used AND whether they are one- or two-sided<br>*Only common tests should be described solely by name; describe more complex techniques in the Methods section.*
☒	☐	A description of all covariates tested
☒	☐	A description of any assumptions or corrections, such as tests of normality and adjustment for multiple comparisons
☐	☒	A full description of the statistical parameters including central tendency (e.g. means) or other basic estimates (e.g. regression coefficient) AND variation (e.g. standard deviation) or associated estimates of uncertainty (e.g. confidence intervals)
☐	☒	For null hypothesis testing, the test statistic (e.g. *F*, *t*, *r*) with confidence intervals, effect sizes, degrees of freedom and *P* value noted<br>*Give P values as exact values whenever suitable.*
☒	☐	For Bayesian analysis, information on the choice of priors and Markov chain Monte Carlo settings
☒	☐	For hierarchical and complex designs, identification of the appropriate level for tests and full reporting of outcomes
☐	☒	Estimates of effect sizes (e.g. Cohen's *d*, Pearson's *r*), indicating how they were calculated

Our web collection on statistics for biologists contains articles on many of the points above.

Software and code

Policy information about availability of computer code

Data collection	1. ThT fluorescence values were collected using SF6 software, version 5.12.1, Corona Electric Co,LTD, Japan<br>2. CD spectra were collected using Spectra Manager V. 1.55.00, JASCO Corp.<br>3. Chromatographic data were collected using Clarity V7, DataApex.<br>4. Transmission electron microscopy images were collected using Hitachi H-7650 control software, V. 02.00 0103-03<br>5. Optical density of the samples in ELISA assay for quantification of serum b2m concentration was collected using SF6 software, version 5.12.1, Corona Electric Co,LTD, Japan.<br>6. QCM data were collected by a laboratory-developed software.<br>7. Nuclear magnetic resonance experimental (NMR) data were collected employing TOPSPIN-NMR software V. 4.1.1, Bruker Biospin.<br>8. Fluorescence spectrum of ThT fluorescence was collected using FL solutions version 4.2, Hitachi.
Data analysis	All data analyses were performed using Sma4 (ver. 1.58), Microsoft Excel 2019 MSO (Microsoft), and Matlab R2020b (Mathworks). For NMR data, the analysis was performed using Sparky (ver. 3.114). All molecular illustrations were creased using PyMOL Molecular Graphics (ver. 2.5.1).

For manuscripts utilizing custom algorithms or software that are central to the research but not yet described in published literature, software must be made available to editors and reviewers. We strongly encourage code deposition in a community repository (e.g. GitHub). See the Nature Portfolio guidelines for submitting code & software for further information.

Data

Policy information about availability of data

All manuscripts must include a data availability statement. This statement should provide the following information, where applicable:

- Accession codes, unique identifiers, or web links for publicly available datasets
- A description of any restrictions on data availability
- For clinical datasets or third party data, please ensure that the statement adheres to our policy

The experimental data (Figs. 1, 2, 3, 4, and 5, and Supplementary Figs. 3, 5, 6, 7, 8, 10, 11, and 12) generated in this study are provided in Supplementary Data 2.

Field-specific reporting

Please select the one below that is the best fit for your research. If you are not sure, read the appropriate sections before making your selection.

[x] Life sciences [ ] Behavioural & social sciences [ ] Ecological, evolutionary & environmental sciences

For a reference copy of the document with all sections, see nature.com/documents/nr-reporting-summary-flat.pdf

Life sciences study design

All studies must disclose on these points even when the disclosure is negative.

Sample size	Sample size was determined based on study regarding dialysis-related amyloidosis: DOI: 10.1007/s10157-021-02122-8.
Data exclusions	In the HANABI assays (Supplementary Figure 7 and 8), we excluded data acquired in wells where the ultrasonic intensity was not enough because of the deterioration of the ultrasonic transducers. For this data exclusion, we identified wells to be excluded before a series of experiments, and data obtained in the wells were excluded for all data in a series of experiments. Moreover, we retried the ThT measurement of samples which did not fulfill the following criteria: (i) The six standard solutions without human sera (i.e., internal standard) showed an increase in the ThT fluorescence intensity; and (ii) The CV value of the lag time among triplicates or more is less than 25%. In other assays, no data were excluded.
Replication	All the measurements were performed by triplicate or more. All the replicates are included in the data. Except for the HANABI assays mentioned above, all attempts at replication were successful.
Randomization	Because this study is the cross-sectional observational study, the patient groups were clearly divided based on the history of dialysis treatment. Thus, the randomization was not applicable in this study.
Blinding	In our experimental protocol, the person who collected serum samples labeled the ID of samples and send the person who performed the experiments without disclosure of the patient information. After the series of the HANABI assay, the person who performed the experiment is informed which patient belongs to which group and started to analyze the experimental results. This blinding protocol was adopted to avoid the intervention of prejudices of the person in charge of the experiment.

Reporting for specific materials, systems and methods

We require information from authors about some types of materials, experimental systems and methods used in many studies. Here, indicate whether each material, system or method listed is relevant to your study. If you are not sure if a list item applies to your research, read the appropriate section before selecting a response.

Materials & experimental systems

n/a	Involved in the study	
[x]	[ ]	Antibodies
[x]	[ ]	Eukaryotic cell lines
[x]	[ ]	Palaeontology and archaeology
[x]	[ ]	Animals and other organisms
[ ]	[x]	Human research participants
[x]	[ ]	Clinical data
[x]	[ ]	Dual use research of concern

Methods

n/a	Involved in the study	
[x]	[ ]	ChIP-seq
[x]	[ ]	Flow cytometry
[x]	[ ]	MRI-based neuroimaging

Human research participants

Policy information about studies involving human research participants

Population characteristics	Because we treated 118 sera from 88 patients in this study, we provided the detailed clinical information for identical patients and population characteristics of patient cohorts in Supplementary Data 1. The file includes patient information such as age, gender, concentrations of serum components, status of dialysis treatment, and cause of kidney disease.
Recruitment	We recruited donors of serum samples by informing the purpose of this study and collected serum samples from donors who agreed with the use of samples for this research; 30 non-dialysis controls in a single center and 58 patients undergoing long-term dialysis treatment in multi-centers. As we provided in Supplementary Data 1, there is no bias to declare in the individuals treated in this study.
Ethics oversight	This study complies with all relevant ethical regulations. The study protocol adhered to the Declaration of Helsinki and was approved by the Central Ethics Committee of Niigata University (2018-0054/ 2022-0019) and Osaka University (T21069). All patients provided written informed consent. An opt-out option was also provided to allow patients to refuse study participation.

Note that full information on the approval of the study protocol must also be provided in the manuscript.
